# Engineering Organic Molecule–Copper Interfaces for Selective C_2+_ Production in CO_2_ Electroreduction

**DOI:** 10.3390/ma19194039

**Published:** 2026-09-22

**Authors:** Qianhui Feng, Keying Cao, Gan Qu, Minhan Li

**Affiliations:** College of Materials Science and Engineering, Zhengzhou University, Zhengzhou 450001, China; 18746490972@163.com (Q.F.); 18436325092@163.com (K.C.); gqu@zzu.edu.cn (G.Q.)

**Keywords:** CO_2_ electroreduction, Cu-based catalysts, organic–Cu interfaces, molecular modification, interfacial microenvironment, electronic-structure regulation, C–C coupling, C_2+_ products

## Abstract

**Highlights:**

- This review summarizes the engineering of organic molecule–Cu interfaces for CO_2_RR.
- The types of modified molecules and their interactions with Cu surfaces are discussed in detail.
- Dynamic interfacial stability is assessed under realistic electrochemical operating conditions.
- The promotion mechanism of organic-inorganic interfaces on CO_2_RR performance is systematically summarized.
- The future opportunities are proposed for selective, durable, and scalable CO_2_-to-C_2+_ electrolysis.

**Abstract:**

The electrochemical carbon dioxide reduction reaction (CO_2_RR) serves as a crucial bridge linking CO_2_ utilization, renewable electricity, and chemical production. Driven by intermittent clean energy sources such as wind and solar power, it can convert CO_2_ into high-value chemicals and fuels under ambient temperature and pressure and is therefore regarded as a promising route for artificial carbon recycling and sustainable manufacturing of chemicals. Copper (Cu) is currently the only metal capable of deep CO_2_ reduction to multicarbon (C_2+_) products in aqueous electrolytes. However, the product selectivity and catalytic stability of Cu-based catalysts still demand further improvement. In recent years, organic molecule modification strategies have emerged as an effective approach to optimizing Cu-based CO_2_RR catalysts and have attracted increasing attention. Organic molecules can form favorable organic-inorganic interfaces with the Cu surface through various interactions, promoting the formation of C_2+_ products via multiple mechanisms. This review summarizes the recent advances in constructing efficient Cu-based catalysts for CO_2_RR through organic molecule modification strategy. By reviewing the fundamentals for C_2+_ formation over Cu-based catalysts, this paper first sorts out the interfacial structures and interactions between the organic molecules and Cu surfaces. Then, the underlying mechanisms of the well-defined organic molecule–Cu interfaces for enhanced CO_2_RR performance are discussed, including the modulation of surface charge and oxidation state of Cu, the tuning of the local microenvironments, and the synergies for activating CO_2_ or stabilizing key intermediates. Furthermore, current research challenges and future prospects associated with the engineering of organic molecule–Cu interfaces are outlined, aiming to provide insights for the rational design of Cu-based catalysts for CO_2_RR.

## 1. Introduction

Give the intensifying global climate change and the continued pursuit of carbon-peaking and carbon-neutrality goals, the efficient valorization of carbon dioxide (CO_2_) has become a major scientific challenge shared by the energy, chemical, and materials communities [1]. As an important bridge between renewable electricity and chemical production, the electrochemical CO_2_ reduction reaction (CO_2_RR) can be driven by intermittent clean energy sources such as wind and solar power to convert CO_2_ into value-added chemicals and fuels, including carbon monoxide, methane, ethylene, and ethanol, under ambient temperature and pressure [2]. Accordingly, CO_2_RR is widely regarded as a key technological route toward carbon recycling and sustainable chemical manufacturing [3]. In recent years, advances in electrolyzer design, catalyst development, and reactor engineering have enabled increasingly stable CO_2_ electrolysis at industrially relevant current densities, further accelerating the transition of CO_2_RR from fundamental research toward practical application [4,5].

CO_2_RR can produce a variety of high-value products ranging from carbon monoxide and formic acid to methane, ethylene, ethanol and propanol [2]. Their reaction pathways are complex, involving multiple electron and proton coupling transfer processes. Thus, the catalyst’s control over the adsorption behavior and transformation of key intermediates play a key role in determining the reaction’s activity and selectivity of CO_2_RR [6]. Among the metal electrocatalysts reported to date, different metals exhibit distinct product preferences, noble metals such as Au and Ag primarily generate CO, while metals like Sn, Bi, and In tend to produce formic acid (or its salts), whereas most transition metals struggle to achieve efficient CO_2_ reduction due to competition from the hydrogen evolution reaction (HER) [1,2]. In contrast, Cu is currently the only metal catalyst capable of deeply reducing CO_2_ to C_2+_ products in aqueous electrolytes and has therefore attracted considerable attention in the CO_2_RR research community [2]. In the 1980s, the pioneering work of Hori and co-workers systematically revealed the product distributions of CO_2_RR on different metal electrodes and established the unique ability of Cu to generate value-added products such as methane, ethylene, and ethanol, thereby laying the foundation for modern Cu-based CO_2_ electroreduction research [7,8,9]. The distinctive catalytic behavior of Cu arises fundamentally from its moderate binding strength toward key reaction intermediates and its unique surface electronic structure. Adsorbed CO (*CO) is a key intermediate in the formation of deeply reduced products such as methane, ethylene, and ethanol. On Au and Ag, CO binds too weakly and readily desorbs, preventing its subsequent hydrogenation and C–C coupling. However, Ni and Pt bind CO too strongly, causing persistent surface occupation and catalyst poisoning [10]. In contrast, Cu can stabilize CO with moderate binding strength, preventing it from either desorbing rapidly or occupying the active site for extended periods, thereby creating the necessary conditions for subsequent proton–electron transfer and C–C coupling [2,11,12]. This moderate interaction with *CO is a primary reason why Cu is uniquely capable of efficiently producing C_2+_ products in aqueous electrolytes. More recently, density functional theory (DFT) calculations and in situ/operando spectroscopic investigations have provided deeper insights into the reaction mechanism of Cu-based CO_2_RR. These studies reveal that the moderate adsorption of *CO and the facet-dependent stabilization of reaction intermediates are critical factors governing the formation pathways of different products. Such mechanistic understanding provides a theoretical foundation for the rational design of advanced Cu-based catalysts [13,14,15].

Based on these mechanistic insights, various strategies have been proposed to enhance the catalytic performance of Cu-based CO_2_RR catalysts [16]. These approaches mainly focus on regulating the electronic structure, surface configuration, and intermediate adsorption behavior through facet engineering, alloying, oxide-derived structure construction, and nanoscale morphology control [2,14,15,17]. These approaches can, to some extent, tune *CO adsorption, alter the local reaction environment, and improve C_2+_ selectivity, thereby substantially advancing CO_2_RR performance [2,18,19]. However, most conventional strategies regulate the intrinsic properties of the metal only indirectly, offer a relatively limited design space, and often involve trade-offs among selectivity, current density, and stability [5]. Consequently, despite major advances achieved at laboratory-scale, Cu-based catalysts are still facing great challenges, like poor, selectivity, inferior long-term stability, and unclear reaction mechanism [20,21]. This limitation indicates that optimization based solely on the bulk structure of the metal is approaching a practical bottleneck and highlights the need for interfacial engineering strategies that extend beyond conventional control of morphology, composition, and electronic properties.

Recent studies have increasingly demonstrated that CO_2_RR performance is governed not only by the electronic and geometric properties of the catalyst surface but also by the local reaction environment at the electrode/electrolyte interface. Under operating conditions, the catalytic interface develops a microenvironment that can differ markedly from the bulk solution, including local CO_2_ enrichment, pH gradients, ion redistribution, and changes in solvation structure [22,23,24]. These factors collectively influence the formation, stabilization, and subsequent conversion of key intermediates such as *CO_2_^−^, *CO, and *CHO, and therefore strongly affect the final product distribution [2,25,26,27,28]. A growing body of experimental and theoretical evidence indicates that regulation of the intrinsic electronic structure alone is insufficient to regulate catalytic performance and understand the mechanism of CO_2_RR, while the interfacial microenvironment engineering represents an additional and increasingly important dimension for CO_2_RR [22,23]. Although the decisive role of the interfacial microenvironment has been proposed, its precise and programmable design remains a central challenge. Traditional inorganic regulation strategies, such as facet engineering, alloying, and nanostructuring, mainly influence the interfacial reaction environment indirectly through changes in the catalyst itself and therefore offer limited molecular-level control over local electric fields, solvation ions, and intermediate stabilization [4,20]. From a broader perspective, an early and influential demonstration of molecular-scale regulation of electrochemical CO_2_ reduction was reported by Rosen et al., who showed that an imidazolium-based ionic liquid could strongly alter CO_2_ reduction on Ag and substantially decrease the required overpotential [29]. Molecular-interface construction strategies, including electrografting, self-assembled monolayers, and polymer coatings, were subsequently developed to provide chemically and structurally tunable electrode interfaces [30,31]. Their implementation in Cu-based CO_2_RR—including amino-acid modifiers, molecular films, hydrophobic molecular layers, polyamine coatings, and poly(ionic liquid) interfaces—has established a rapidly growing area of molecular interfacial engineering [32,33,34]. In recent years, organic interfacial engineering has attracted particular attention because the chemical identity, anchoring group, molecular dipole, steric structure, hydrophobicity, and ionic functionality of organic modifiers can be tailored at the molecular level. This tunability enables simultaneous regulation of Cu electronic properties and the surrounding reaction microenvironment, including intermediate adsorption, local electric fields, interfacial water structure, proton transfer, and reactant availability [35,36,37]. Recent reviews have summarized the participation of organic molecules in Cu-based CO_2_RR and highlighted the ability of organic modifiers to regulate both intrinsic catalyst properties and the local reaction environment [35,36,37]. However, these systems encompass substantially different interfacial architectures-from directly adsorbed molecular modifiers and polymeric or ionic layers to ligand-defined Cu coordination environments-and a unified comparison of their interfacial structures, mechanistic functions, stability, and applicability under practically relevant conditions remains limited [35,36,37,38]. In particular, the distinction between genuine organic–Cu interfaces and conceptually related systems have not always been made explicit, making it difficult to directly compare the mechanistic roles of different interfacial regulation strategies. Therefore, a mechanistically organized framework that connects interface construction, interfacial structure, catalytic function, and operational stability is needed. Such an understanding is important not only for the rational design of Cu catalysts toward selective C_2+_ electrosynthesis but also for developing more general principles for controlling catalyst–electrolyte interfaces in electrochemical transformations.

Accordingly, the optimization of Cu-based CO_2_ electroreduction has gradually evolved from isolated control of catalyst structure toward coordinated regulation of the catalyst–electrolyte interface at the molecular scale. In this review, the term organic–Cu interface primarily refers to interfaces in which organic molecules, ligands, polymers, or ionic species directly interact with Cu surfaces or Cu active sites through chemical bonding, coordination, electrostatic interactions, hydrogen bonding, π interactions, or other noncovalent interactions. These directly modified interfaces constitute the central scope of this review. Coordination polymers and organic framework-confined Cu sites are discussed separately as extended organic-coordination environments, in which the organic ligand field forms an integral part of the local Cu active-site structure rather than functioning simply as a molecular overlayer on a metallic Cu surface. In contrast, sulfur-modified Cu, Cu sulfide-derived catalysts, and intrinsically hydrophobic Cu electrodes are not classified as organic–Cu interfaces in this review; they are included only selectively as mechanistic reference systems when they provide useful insight into specific effects such as Cu–S electronic coupling, surface reconstruction, or hydrophobic microenvironment regulation. Rather than simply categorizing organic modifiers according to their chemical identities, this review organizes organic–Cu interface engineering according to the interfacial interactions, active-site structures, and catalytic functions established at Cu. First, summarize the fundamental reaction network and the origin of C_2+_ selectivity on Cu surfaces. Then discuss strategies for constructing organic–Cu interfaces, including chemically bonded and coordinated molecular interfaces, noncovalently assembled interfaces, polymer coatings, and ionic-liquid-based systems, together with their stability under electrochemical conditions. Subsequently, the mechanisms by which these interfaces regulate Cu electronic structure, local reaction microenvironments, intermediate stabilization, and cooperative catalytic pathways are critically examined. Particular attention is paid to distinguishing different interface architectures, comparing their advantages and limitations, and identifying unresolved questions concerning active-site identity, structure–function relationships, interfacial stability, and the transferability of mechanistic conclusions from model conditions to practically relevant CO_2_ electrolysis. Finally, current knowledge gaps and future opportunities for developing molecularly defined, mechanistically understood, and operationally stable organic–Cu interfaces are discussed.

## 2. Reaction Networks and Selective for C_2+_ Production on Cu Surfaces

The electrochemical CO_2_RR, which converts CO_2_ into value-added fuels and chemicals, is an important component of renewable-electricity-driven carbon cycling. However, the high thermodynamic stability of CO_2_ and the low electron affinity associated with its linear molecular structure make activation kinetically demanding and lead to complex multielectron and proton-coupled reaction pathways. As summarized in Figure 1, CO_2_ electroreduction on Cu involves a highly branched reaction network comprising competing pathways toward CO, formate/formic acid, methane, methanol, and C_2+_ products. The formation of C_2+_ products requires the accumulation and coupling of surface-bound carbon intermediates, whereas the final product distribution is determined by the subsequent proton–electron-transfer sequence, intermediate adsorption configuration, and local reaction environment. This overall reaction framework provides the mechanistic basis for the following discussion of CO_2_ activation, C–C coupling, and structure-dependent selectivity on Cu [1,39].

### 2.1. CO_2_RR Activation on Cu and the Origin of C_2+_ Selectivity

CO_2_RR proceeds through multiple electron-transfer and proton-coupled steps, with the reaction pathways depending on intermediate adsorption, interfacial kinetics, and surface structure. Among reported metal electrocatalysts, Cu is a distinctive system that can generate C_2+_ products with appreciable selectivity under aqueous conditions [11]. In contrast to Au and Ag, which predominantly produce CO, Cu binds *CO sufficiently strongly to retain CO-derived intermediates on the surface for subsequent reduction [11,40]. A sufficient surface *CO coverage provides favorable conditions for C–C coupling, while excessively strong adsorption can hinder the subsequent conversion of adsorbed intermediates [41,42]. As shown in Figure 2a, the binding energies of *CO and H can be used to classify metal catalysts according to their dominant CO_2_RR products [40]. Cu is distinguished from many other metals by its combination of favorable CO binding and relatively weak H adsorption, which favors further CO conversion while limiting competition from the hydrogen evolution reaction [40].

The initial activation of CO_2_ generally involves conversion of linear CO_2_ into a bent CO_2_^−^ configuration, a high-energy process that is considered one of the kinetically critical steps in CO_2_RR [43]. On Cu, CO_2_ undergoes electron transfer and proton-coupled conversion to a *COOH intermediate, followed by dehydration to form *CO, as schematically illustrated in Figure 2b. *CO therefore connects the initial activation of CO_2_ with subsequent C–C coupling [14]. The moderate CO binding strength of Cu allows a sufficient surface coverage to be maintained for C–C coupling, whereas weaker binding leads to rapid CO desorption and stronger binding hinders subsequent conversion [11,43]. Studies have shown that efficient C_2+_ formation on Cu requires the accumulation of a high local coverage of *CO and its subsequent coupling to form a C–C bond [41]. An appropriate combination of CO adsorption strength and surface structure lowers the barrier for C–C coupling. Thus, the distinctive CO adsorption characteristics and C–C coupling capability of Cu constitute the fundamental origin of its unique C_2+_ selectivity.

Nevertheless, C_2+_ formation on Cu is not governed solely by the intrinsic properties of the metal. The selectivity is also influenced by crystal orientation, local electronic states, surface reconstruction, and reaction conditions [44]. These factors jointly determine competition among the reaction pathways on Cu and provide the mechanistic basis for further selectivity control through interface engineering.

### 2.2. Mechanism for C–C Coupling and C_2+_ Product Formation

In Cu-catalyzed CO_2_RR, *CO is the key intermediate linking CO_2_ activation to C_2+_ product formation. CO_2_ is converted through successive proton–electron transfer steps to *COOH and then to *CO. When the Cu surface sustains an adequate *CO coverage, adjacent *CO species can undergo coupling, providing the prerequisite for C–C bond formation [41].

The most widely accepted C–C coupling mechanism is *CO dimerization, in which two *CO molecules interact through their carbon atoms to form the key *OCCO intermediate. Theoretical calculations and in situ spectroscopy indicate that *OCCO formation is a critical step in C_2+_ production on Cu and that its stability strongly affects subsequent pathways [45,46]. Two adsorbed *CO species evolve through a dimerization transition state to form *OCCO. The calculated coupling barrier is 0.33 eV on Cu(100), substantially lower than the 0.68 eV value on Cu(111), demonstrating that Cu(100) is more favorable for *CO dimerization and C–C bond formation [47]. Other coupling routes have also been proposed, including CO insertion and pathways involving *CHO. These mechanisms generally depend on specific surface structures and operating conditions and remain less universally accepted than direct *CO dimerization [48]. Under selected potentials and surface configurations, *CO may first be hydrogenated to *CHO and subsequently couple with another CO to form COCHO [48]. Subsequent proton–electron transfer at different sites can lead to ethylene, ethanol, acetate, and other C_2_ oxygenates [41,44].

Following formation of *OCCO, successive proton–electron transfers and removal of oxygen-containing species lead to branching pathways toward ethylene, ethanol, propanol, and other C_2+_ products [49,50]. The distribution among these products is primarily determined by the subsequent conversion sequence of adsorbed intermediates and by the reaction conditions [51]. Overall, the ability of Cu to retain an appropriate *CO coverage while avoiding excessively strong adsorption provides the basis for C_2+_ formation [11,43]. Overall, the central requirement for C_2+_ formation on Cu is the effective accumulation of *CO and subsequent C–C coupling, while surface structure, electronic state, and interfacial environment further determine the final product distribution. This mechanistic framework provides the basis for catalyst-structure optimization and interface engineering [2,14].

### 2.3. Key Factors Governing the CO_2_RR Performance on Cu-Based Catalysts

In addition to the reaction pathways and intermediate evolution described above, the structural characteristics of Cu surfaces are critical determinants of C_2+_ selectivity [2,15]. Exposed facets, atomic arrangements, and surface defects alter the adsorption configurations and reaction barriers of key intermediates and thereby redirect CO_2_RR pathways [15,41,44]. For example, Cu(100) generally exhibits strong C–C coupling ability and favors C_2_ products such as ethylene, whereas Cu(111) displays different CO adsorption and conversion characteristics and tends toward a different product distribution [8,41,43]. Control over Cu nanocrystal morphology and facet exposure can therefore optimize C_2+_ selectivity [15,41]. As shown in Figure 2c, the C_2_H_4_/CH_4_ selectivity ratio varies markedly with crystallographic orientation and step structure. Cu(100) and (100)-type surfaces containing suitable amounts of (111) or (110) steps show high ethylene selectivity, whereas close-packed surfaces such as Cu(111) favor C_1_ products [2,52,53].

Cu catalysts also undergo dynamic surface reconstruction under operating conditions. The oxidation state, defect population, and local coordination environment evolve with potential and consequently affect intermediate adsorption and reaction pathways. Oxidized Cu species, undercoordinated sites, and structural defects may influence C_2+_ formation by tuning CO adsorption and intermediate stabilization [54]. Understanding the working-state structure of Cu under realistic reaction conditions is therefore essential for identifying the origin of selectivity. The dense Cu_2_O cubes can transform at CO_2_RR potentials into fragmented, porous Cu structures, accompanied by dissolution, redeposition, and aggregation of small Cu nanoparticles, demonstrating that the active catalyst differs substantially from the as-prepared material [55,56].

Electrolyte composition and operating conditions likewise influence the local reaction environment and product distribution on Cu. In particular, Alkali-metal cations can promote C–C coupling through coupled electrostatic and solvation effects at the electrode–electrolyte interface. Under cathodic polarization, cations accumulate within the electrical double layer and modify the potential drop and electric-field strength across the Stern layer. Because several CO_2_RR intermediates, including *CO_2_ and C-C coupling intermediates such as *OCCO, possess substantial dipole moments and polarizabilities, stronger interfacial electric fields can preferentially stabilize these polar adsorbates and lower the energetic penalty associated with CO_2_ activation and C–C bond formation, thereby favoring C_2+_ products [57,58]. Alkali-metal cations can also directly interact with adsorbed *CO species; for example, specifically adsorbed K^+^ near *CO induces interfacial charge redistribution and lowers the *CO-*CO coupling barrier, further facilitating multicarbon formation [59]. In addition, cations reorganize the interfacial water network and thereby regulate proton accessibility to adsorbed intermediates. Recent in situ spectroscopic studies showed that K^+^-associated interfacial water can disrupt the hydrogen-bond network around *CO while simultaneously stabilizing adjacent *CO species, suppressing their hydrogenation toward C_1_ products and increasing the probability of C-C coupling toward C_2_ products [60]. Current density, applied potential, and mass-transport conditions further modify the local pH, reactant availability, and intermediate coverage. Therefore, coordinated optimization of catalyst structure, electrolyte composition, and operating conditions is essential for achieving high C_2+_ selectivity on Cu-based catalysts.

Taken together, the preceding discussion demonstrates that C_2+_ selectivity on Cu is governed by a coupled set of factors, including surface atomic structure, oxidation-state evolution, intermediate coverage, interfacial electric fields, solvation structure, and mass transport. Conventional strategies based on facet control, defect engineering, and optimization of electrolyte or operating conditions can influence these factors, but they generally do so indirectly and offer limited molecular-level precision. Organic modifiers provide a complementary approach because their anchoring groups, functional moieties, charge states, and assembly structures can be rationally designed to regulate both the Cu surface and the surrounding reaction microenvironment. The following section therefore shifts from the intrinsic reaction characteristics of Cu to the deliberate construction of organic–Cu interfaces, focusing on how covalent grafting, noncovalent adsorption, polymer coatings, and ionic-liquid layers establish structurally defined platforms for regulation of CO_2_RR performance on Cu-based materials [32,61,62,63].

## 3. Strategies for Engineering Organic Interfaces on Cu Surfaces

Building on the mechanistic framework established above, this section examines how organic modifiers can be used to deliberately engineer Cu/electrolyte interfaces. The discussion is organized according to the dominant mode of molecule–Cu interaction, including covalent grafting, noncovalent adsorption, and polymer- or ionic-liquid-based interfacial modification. Particular emphasis is placed on the structural characteristics, formation mechanisms, and stability of these interfaces, thereby establishing the structural foundation for the subsequent analysis of how organic modifiers regulate CO_2_RR activity and product selectivity.

### 3.1. Organic Molecular Modification and Interactions with Cu Surfaces

The central objective of organic modification of Cu-based catalysts is to use chemically defined functional groups to construct organic–metal interfaces with controllable structural characteristics. Unlike conventional alloying, defect engineering, or facet control, organic modification generally leaves the Cu lattice largely unchanged and instead introduces a designable surface layer through chemical bonding, coordination, or noncovalent interactions between the molecule and the metal [62]. On the basis of the binding mode, these interfaces can be broadly divided into chemically bonded/coordinated and noncovalently adsorbed systems. The former rely on specific functional groups to establish stable connections with the Cu surface, such as Cu–C [62] and Cu–S [61] interactions, whereas the latter employ weaker interactions including π–metal interactions, electrostatics, and hydrogen bonding [32]. These binding modes differ in interfacial stability, molecular arrangement, and structural tunability and therefore provide complementary foundations for constructing functional organic–Cu interfaces [63]. The representative modes of organic molecular modification and their interactions with Cu surfaces are schematically summarized in Figure 3.

#### 3.1.1. Stable Organic–Cu Interfaces Constructed Through Chemical Bonding and Coordination

The chemical bonding and coordination are major routes for constructing stable organic–Cu interfaces. Specific functional groups form relatively strong interactions with surface Cu atoms or active sites, thereby immobilizing the molecular layer on the metal. Depending on the anchoring mode, representative interfaces include Cu–C covalent bonds, Cu–S linkages, and Cu–N coordination. Compared with simple physisorption, these interactions generally provide higher binding strength, improve the stability of organic layers in electrochemical environments, and enable structurally programmable organic–Cu interfaces.

The electrochemical grafting of aryldiazonium salts is a representative method for constructing aryl-derived layers on conductive substrates. During electroreduction, aryldiazonium species generate reactive aryl radicals that subsequently react with the electrode surface, as illustrated in Figure 4a [30]. This strategy can provide persistent and structurally tunable organic layers. However, when it is applied to Cu, the formation and chemical identity of a direct Cu–C bond should be verified for each specific system rather than inferred solely from the general electrografting mechanism.

Cu–S interactions are also widely used to construct organic layers because they combine relatively strong binding with controllable molecular organization. Thiol modification is among the most extensively employed approaches for organic functionalization of metal surfaces. Owing to the high affinity of sulfur for metals, thiols can bind to Cu through Cu–S linkages and form partially ordered SAMs, as schematically shown in Figure 4b [31]. Varying the alkyl-chain length, terminal functional group, and molecular packing changes the chemical and structural characteristics of the Cu interface, making the Cu–S linkage both an anchoring motif and a platform for interfacial regulation. As a distinct sulfur-modified rather than thiol-SAM example, Shinagawa and co-workers prepared sulfur-modified Cu by in situ reductive reconstruction of a nanoscale CuS precursor. The resulting catalyst maintained a formate Faradaic efficiency of approximately 80% at −0.8 V vs. RHE while suppressing the characteristic CO pathway and C_2+_ formation on Cu. XPS and XANES analyses (Figure 4c) showed that residual surface sulfur altered the local electronic environment of Cu and weakened Cu–CO interactions, thereby modifying intermediate adsorption during CO_2_RR [64].

N-containing organic modifiers can interact with Cu-based surfaces and alter the local adsorption environment and reaction energetics. Pyridine-modified CuO/Cu(OH)_2_ has been reported as an example of N-containing surface modification [65]. However, the presence of pyridine-derived species at the surface should not be described as direct structural proof of a well-defined Cu–N coordination bond unless such coordination is independently verified by XAS or related structural characterization. Oxygen-containing anchoring groups, including phosphonate and carboxylate moieties, can provide stable attachment to hydroxylated or oxide-containing surfaces. Organophosphonate layers have been investigated on Cu-related interconnect surfaces, supporting their relevance to Cu surface functionalization. However, because Figure 4d presents a ZnO model comparing thiol and phosphonic-acid anchoring groups, it should therefore be used only to illustrate general oxide-surface anchoring principles [66,67]. Its results should not be regarded as direct evidence for an O-bridged organic–Cu interface under CO_2_RR conditions.

These oxygen-anchoring strategies have potential in oxide-derived Cu catalysts. However, because Cu surfaces undergo dynamic redox transformations during CO_2_RR, the stability of O-bridged interfaces and their influence on reaction pathways require further investigation.

#### 3.1.2. Dynamic Organic–Cu Interfaces Constructed Through Noncovalent Interactions

The dynamic organic interfaces on Cu can arise either from reversible noncovalent adsorption or from the in situ formation of persistent organic films, such as N-arylpyridinium-derived films electrochemically formed on Cu [33,34,35]. These interfaces may reorganize in response to electrode potential, electrolyte composition, competitive adsorption, and surface reconstruction. Rather than necessarily involving direct covalent bonding to Cu, molecular retention at such interfaces can be governed by π-metal interactions, electrostatic attraction, hydrogen bonding, and van der Waals forces. Because these interactions are relatively weak and reversible, the interface retains substantial structural flexibility and can undergo molecular rearrangement as the electrochemical environment changes, thereby reaching a dynamically stabilized configuration [63,68].

The π–Metal interactions can contribute to the adsorption of aromatic molecules on Cu surfaces, particularly when the conjugated molecular framework adopts a near-parallel orientation that maximizes its contact with the metal [69]. Such adsorption generally arises from a combination of dispersion forces and electronic coupling between the molecular π states and the electronic states of surface Cu atoms [70]. Consequently, the interfacial interaction is sensitive to the size and aromaticity of the conjugated framework, the identity and electronic character of its substituents, and the crystallographic structure of the Cu surface. Electron-donating or electron-withdrawing groups can alter interfacial charge redistribution and adsorption geometry, whereas an extended aromatic framework can increase the molecule–surface contact area [69,70]. At higher surface coverage, lateral π–π and van der Waals interactions between neighboring molecules further affect packing density, orientation, and domain formation. Because these interfaces are not necessarily immobilized by a single covalent linkage, changes in electrode potential, competitive adsorption of electrolyte species, and reconstruction of the Cu surface may induce variations in molecular orientation and surface coverage. This structural adaptability provides a basis for dynamically responsive organic–Cu interfaces. Nevertheless, π–metal interaction should generally be regarded as one possible contribution to interfacial stabilization rather than assumed to be the exclusive binding mechanism, particularly under electrochemical operating conditions [33,35,69,70].

The electrostatic interactions provide another important route to dynamic organic–Cu interfaces. Charged or ionic organic molecules can accumulate at Cu surfaces and form organic layers with characteristic interfacial structures. Studies of N-substituted pyridinium species indicate that these molecules are not merely physiosorbed during electrolysis; instead, they form an insoluble deposited film on Cu (Figure 5a) [63].The film can restrict proton transport, alter the local pH and surface adsorption environment, increase interfacial CO coverage, and facilitate C–C coupling. The pronounced structure dependence of pyridinium derivatives is reflected in C_2+_/CH_4_ selectivity ratios exceeding 800 for selected aryl-substituted species, demonstrating precise pathway control by a dynamically formed molecular interface (Figure 5b) [34]. Hydrophobic organic molecules can further optimize the local reaction environment by altering Cu wettability. Alkyl chains, fluorinated segments, and other low-surface-energy motifs reduce interfacial water coverage, increase local CO_2_ availability, and impede proton transport required for HER, thereby favoring CO_2_RR.

Flexible polymer layers can likewise form dynamic organic–Cu interfaces through multiple noncovalent interactions. Polymer chains cover Cu through hydrogen bonding, electrostatic interactions, and van der Waals forces, producing an adaptable organic network. Compared with rigid monolayers, polymer interfaces can respond to complex electrochemical conditions through chain rearrangement and conformational changes, providing a strategy for stable yet dynamically adjustable interfaces. Chen and co-workers incorporated polyamines into Cu electrodes and achieved highly selective conversion of CO_2_ to ethylene at −0.47 V vs. RHE in 10 M KOH, with an ethylene Faradaic efficiency of 87 ± 3% and a full-cell energy efficiency of 50 ± 2%, both superior to those of Cu electrodes without polyamine incorporation, for which the ethylene FE was generally below 70%. In situ Raman spectroscopy showed that the polyamine layer increased the surface pH and CO coverage and stabilized key intermediates, indicating that a flexible organic interface can improve local mass transport and proton management while promoting C_2+_ formation [71].

Overall, noncovalently constructed organic–Cu interfaces have lower binding strength than chemically bonded systems but offer greater structural flexibility and dynamic responsiveness. By tuning molecular structure, polarity, and adsorption mode, a wide variety of dynamic interfaces can be generated, providing an important structural foundation for understanding organic-molecule regulation of Cu-based CO_2_RR.

### 3.2. Polymer-Coating and Ionic-Liquid Interfacial Modification

Unlike modification based on individual molecular adsorption or weak coordination, polymer coatings and ionic-liquid interfaces regulate the catalytic interface by constructing a continuous organic layer on Cu. These strategies typically exploit cross-linking among polymer chains, self-assembly of ionic-liquid components, and formation of organic networks to generate relatively stable functional coatings on the metal surface. Depending on the construction method, representative polymer-based interfaces include polymer thin films, polyelectrolyte coatings, and poly (ionic liquid) (PIL) layers. Compared with single-molecule modifiers, polymer and ionic-liquid interfaces offer more complete surface coverage and greater structural tunability, allowing systematic design through variation in chain architecture, functional groups, and ionic-liquid composition. They also reduce the rapid migration of individual molecules under electrochemical conditions and thereby provide a route to long-lived organic–Cu interfaces [72].

#### 3.2.1. Interfacial Regulation by Polymer Coatings

Polymer-coating modification constructs a continuous organic layer on Cu using macromolecular materials. Compared with small molecules, polymers have larger dimensions, multifunctional chain segments, and tunable spatial architectures, enabling more stable interfaces through interchain interactions and multipoint binding. Polymer systems used in CO_2_ electroreduction include ionomers, polyelectrolytes, and functional-group-containing polymers. Nafion, polyamines, and PILs are among the most widely studied because of their film-forming ability and structural designability.

Su and co-workers systematically investigated the effect of Nafion loading on CO_2_RR over Cu and found that an appropriate coating amount effectively regulated the local Cu/electrolyte environment. At an optimized Nafion loading of 30 μL (Cu@Nafion-4), the C_2_-product Faradaic efficiency reached 73.5% at −1.2 V vs. RHE, approximately 2.6 times that of bare Cu (28.3%), while the HER Faradaic efficiency decreased from 40.6% to 16.8%. The enhancement was attributed primarily to the hydrophobic PTFE backbone of Nafion, which restricts water transport and suppresses HER, together with Lewis acid–base interactions between fluorinated groups and CO_2_ that promote interfacial CO_2_ enrichment and activation. The water contact angle increased from 74.1° on bare Cu to approximately 110.4° after Nafion modification, facilitating CO_2_ transport to the catalytic interface. DFT calculations further indicated that Nafion strengthened CO_2_ adsorption from −0.21 to −1.12 eV and lowered the barrier for conversion of CO_2_ to *COOH from 1.41 to 0.51 eV, thereby promoting CO formation and subsequent C–C coupling. As summarized in Figure 6a, the Nafion layer therefore acts not as an inert overlayer but as an interfacial regulator of wettability, CO_2_ availability, and key-intermediate formation, ultimately improving C_2_ selectivity [73].

The polymer coatings have also been applied to oxide-derived Cu catalysts. Zhou and co-workers showed that Nafion converted a hydrophilic Cu(OH)_2_-derived Cu surface into a highly hydrophobic interface; at a Nafion content of 28.4 wt%, the water contact angle reached approximately 140°, favoring a stable gas–liquid–solid triple-phase boundary. In addition, acid–base neutralization between Nafion sulfonic acid groups and Cu(OH)_2_ generated deprotonated anionic sites. Through Donnan exclusion, these sites increased the local interfacial alkalinity and suppressed HER. In a flow electrolyzer, the optimized AN-Cu@Nafion-28.4 wt.% catalyst achieved a total C_2+_ Faradaic efficiency of 83% at 300 mA cm^−2^, including 44% ethylene, compared with 24% ethylene on unmodified Cu(OH)_2_-derived Cu. Concurrently, the H_2_ Faradaic efficiency decreased from 40% to 11%, and the electrode operated stably for 8000 s. DFT calculations indicated that deprotonated Nafion strengthened OH^−^ adsorption from −0.8969 eV on Cu(111) to −1.9578 eV on Nafion–Cu(111) and lowered the *CO dimerization barrier from 1.40 to 0.75 eV. Thus, as illustrated in Figure 6b, Nafion simultaneously regulates wettability, mass transfer, local acid–base chemistry, and C–C coupling on oxide-derived Cu [74].

N-containing polymers have also emerged as important modifiers because their nitrogen-containing functional groups can interact with Cu while regulating the catalytic microenvironment. As shown in Figure 6c, Su and co-workers introduced a p-aminobenzenesulfonic-acid-modified polyaniline (ABSA–PANI) layer between the Cu catalyst and the electrolyte. The Cu/ABSA–PANI electrode achieved a C_2+_ Faradaic efficiency of 81% at a current density of 600 mA cm^−2^ under acidic conditions. The conductive polymer layer balanced the local hydrophobicity, promoted CO_2_ adsorption and activation, and regulated the transport of K^+^, H^+^, and OH^−^ at the electrode–electrolyte interface. Local-pH measurements further demonstrated that the polymer coating substantially altered the interfacial reaction environment during electrolysis (Figure 6c). Functional polymers can therefore serve not only as protective coatings but also as active platforms for regulating mass transport and the catalytic microenvironment of Cu [75]. Low-surface-energy motifs can likewise be introduced to create specially wetting Cu interfaces. The bio-inspired hydrophobic Cu system reported, employed a long-chain alkyl-thiol monolayer rather than a polymer coating; the molecular layer altered surface wettability and markedly changed CO_2_RR selectivity, illustrating how organic molecular design can impart interfacial function [76].

Overall, polymer coatings use macromolecular chain architecture, multiple functional groups, and film-forming capability to construct interfaces distinct from those generated by individual molecules. Compared with small-molecule modifiers, polymer systems generally provide more complete coverage and greater structural stability, while allowing systematic control of interfacial properties through polymer composition and molecular architecture.

**Figure 6:**
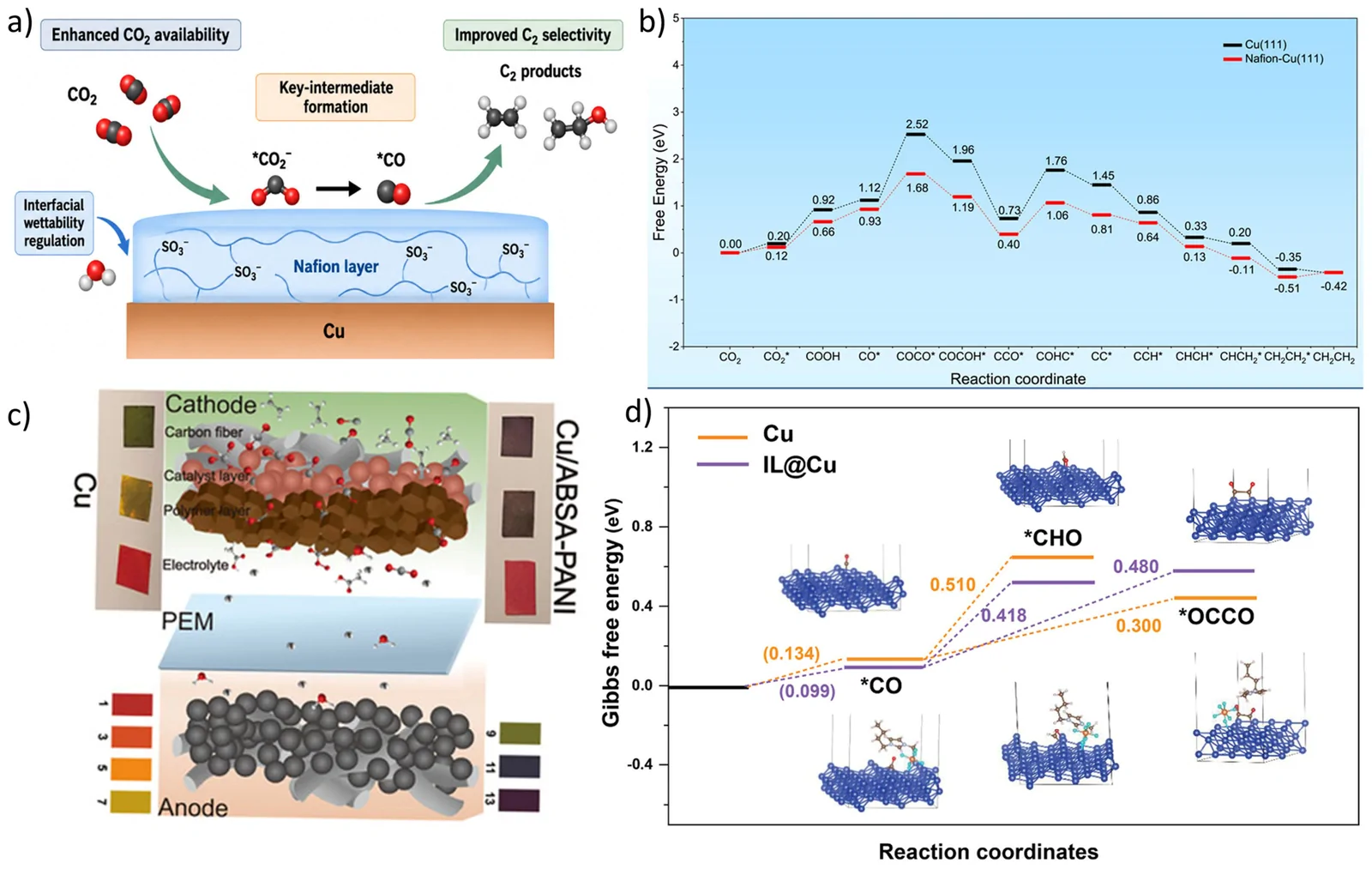
Polymer and ionic-liquid interfaces for regulating the Cu reaction microenvironment. (**a**) Proposed role of a Nafion coating in limiting interfacial water transport, increasing CO_2_ availability, and promoting C_2_-product formation on Cu. (**b**) Calculated free-energy pathways for C_2_H_4_ formation on Cu (111) and Nafion-modified Cu (111). Reproduced under terms of the CC BY 4.0 license [74]. Copyright 2023, Wiley-VCH GMBH. (**c**) Schematic illustration of the Cu/ABSA–PANI electrode configuration for regulating the local microenvironment during acidic CO_2_ electroreduction. The color-bars reflect the pH indicators at corresponding pH values. Reproduced with permission from Ref. [75]. Copyright 2025, Wiley-VCH GmbH. (**d**) Gibbs free-energy comparison of *CO protonation to *CHO and *CO dimerization to *OCCO on bare Cu and ionic-liquid-modified Cu. Reproduced with permission from Ref. [77]. Copyright 2024, Wiley-VCH GmbH.

#### 3.2.2. Interfacial Regulation by Ionic Liquids

The ionic liquids (ILs) are low-melting salts composed of organic cations and inorganic or organic anions. They are characterized by low volatility, high ionic conductivity, and highly tunable structures. Because both the ionic framework and functional groups can be adjusted through molecular design, ionic liquids can serve as specialized organic interfacial materials for functionalizing Cu-based catalysts. Unlike conventional molecular modifiers, ILs form ion-rich organic interfaces on Cu through cation–anion interactions and interfacial assembly.

N-heterocyclic ionic liquids based on imidazolium or pyridinium ions are among the most widely investigated systems in CO_2_RR. Imidazolium ILs in particular combine high CO_2_ affinity with favorable interfacial properties. As shown in Figure 6d, Cai and co-workers modified Cu with [Bmim][PF_6_]. Elemental mapping and XPS showed that the IL was uniformly distributed on the Cu surface and interacted electronically with Cu, altering the local electronic structure [77]. In situ Raman and FTIR measurements further indicated that [Bmim][PF_6_] changed the local proton-donor environment, promoted protonation of *CO to *CHO, and suppressed *CO dimerization to *OCCO, thereby diverting the pathway from C–C coupling toward methane formation. DFT calculations showed that the IL stabilized CO adsorption and lowered the barrier for *CO protonation to *CHO to 0.418 eV. Thus, the imidazolium-derived organic–Cu interface dynamically regulates product selectivity through interfacial charge redistribution, microenvironment modification, and intermediate stabilization [77].

To improve the stability of ionic-liquid interfaces, poly (ionic liquid) (PIL) strategies have been developed. PILs combine the ionic functionality of ILs with the film-forming capability of polymers by fixing ionic-liquid units on a macromolecular backbone, thereby generating a more stable ionic organic network on Cu. Compared with free ILs, PILs exhibit better interfacial retention and greater structural tunability through changes in the polymer backbone, ionic groups, and degree of polymerization. Compared with molecular ionic-liquid–metal interfaces, poly(ionic liquid)–metal interfaces can provide improved interfacial retention while maintaining the ability to regulate CO_2_ transport, anion-mediated interactions, and the local electric field [72]. The preservation of PIL-containing frameworks and Cu-containing domains during prolonged CO_2_RR is separately supported by post-electrolysis XPS, XRD, and SEM characterization [72,78].

The ionomers and PILs that integrate ionic-liquid functionality with polymer frameworks provide an additional route to organic interfaces combining ion transport with mechanical stability. Systematic variation in cation structure, anion identity, and polymer backbone enables diverse interfaces tailored to demanding electrochemical conditions. Ionic liquids and their polymeric derivatives therefore constitute a distinct interfacial-engineering platform for Cu-based CO_2_RR catalysts.

### 3.3. Stability of Molecularly Modified Cu Interfaces

The long-term stability of organic interfaces is a decisive factor in the practical use of molecular modification for Cu-based CO_2_ electroreduction. Unlike conventional inorganic catalyst modifications, organic layers reside at a dynamically evolving electrode/electrolyte interface. Their structural stability depends not only on the initial construction method but also on applied potential, electrolyte composition, and prolonged electrochemical operation. Understanding molecule–Cu binding stability and interfacial evolution under working conditions is therefore essential for sustainable interfacial regulation.

#### 3.3.1. Interfacial Binding Strength and the Basis of Stability

The stability of an organic–Cu interface is determined not only by the initial molecule–metal binding strength but also by the ability of the organic layer to retain its structure during electrolysis. Meaningful stability evaluation must therefore consider resistance to desorption, preservation of interfacial structure, and catalytic-performance decay during long-term operation. Highly cathodic potentials, competitive adsorption of electrolyte ions, and dynamic perturbation of gas–liquid–solid interfaces can induce molecular migration, rearrangement, or desorption during CO_2_RR, making the mode of attachment to Cu a key determinant of durability [79]. In general, stronger interfacial interactions reduce molecular displacement under the combined effects of the electrolyte, electric field, and multiphase-interface fluctuations.

Experimental studies show that organic modifiers capable of forming persistent surface films generally exhibit improved durability. For arylpyridinium-modified Cu, electrochemical reduction generates a poorly soluble oligomeric organic film that remains associated with the Cu surface, as confirmed by XPS and Raman characterization. Moreover, the molecular-film-modified Cu cathode showed robust long-term stability in a membrane-electrode assembly, sustaining a catalytic current of approximately 600 mA and an ethylene Faradaic efficiency of about 60% for nearly 190 h with only minor fluctuations [80]. The persistence and chemical state of the organic film should instead be evaluated on the basis of the corresponding XPS and Raman characterization. Because the available evidence does not unambiguously establish a direct Cu–C covalent bond in this system, its stability is more appropriately attributed to formation and adhesion of the deposited organic layer.

The long-chain thiol modifiers provide a complementary stabilization mechanism. Intermolecular interactions among alkyl chains enhance the integrity of the organic layer, while the sulfur headgroup anchors the molecules to Cu. Octadecanethiol (ODT)-functionalized Cu nanowires retained stable ethylene production during 10 h of electrolysis at 400 mA cm^−2^ demonstrating the durability imparted by Cu–S anchoring and cohesive chain–chain interactions [81]. Nevertheless, strong binding strength is not always optimal. An excessively rigid layer may lose the ability to adapt to the changing electrochemical environment, whereas moderately reversible interactions can permit structural reorganization. Imidazolium-based IL interfaces, for example, are maintained predominantly through ionic and interfacial electrostatic interactions. XPS and elemental analyses show that the IL remains distributed on Cu and continues to influence the reaction environment, illustrating a dynamically stabilized rather than completely static interface [77].

Molecular geometry, functional-group identity, and assembly mode also influence durability. Long-chain molecules increase layer density through van der Waals interactions, whereas multipoint coordination and polymer networks reduce the risk of local desorption by increasing the number of interfacial contacts. For a PIL-modified Cu^0^/Cu^+^ tandem catalyst, N 1s and C 1s signals associated with the PIL backbone remained detectable after a 40 h test, while XRD and SEM showed that the catalyst crystal structure and morphology were largely retained, indicating that a polymeric network can substantially improve long-term interface persistence [78].

Organic–Cu stability is therefore not governed by binding strength alone but by the combined effects of attachment mode, molecular architecture, assembly structure, and dynamic responsiveness. Rational interface design should improve resistance to desorption while preserving sufficient adaptability to achieve a productive balance between stability and function.

#### 3.3.2. Interfacial Evolution and Degradation Under Electrochemical Conditions

The organic modifier layers are not completely static during CO_2_RR but evolve under the electrochemical environment. For weakly adsorbed molecules, changes in potential, electrolyte composition, and competitive adsorption can reorganize the molecular layer; for strongly bound systems, prolonged polarization can still modify interfacial bonds or induce reconstruction of the underlying surface. At the same time, Cu undergoes oxidation-state changes, structural reconstruction, and variations in adsorbate coverage, all of which can alter molecular binding. Figure 7a summarizes representative degradation pathways of Cu-based catalysts, including dissolution, detachment, Ostwald ripening, fragmentation, reshaping, poisoning, and agglomeration [79]. For organic-modified Cu catalysts, these changes in the metal substrate should be considered together with molecular desorption, interfacial rearrangement, ion exchange, and chemical degradation of the organic layer. Electrochemical polarization continuously changes the charge state, adsorbate coverage, and interfacial solvation structure of Cu, causing corresponding adjustments in molecular arrangement and binding. Long-term stability therefore depends not only on the initially assembled interface but also on its evolution under operating conditions. Surface reconstruction, changes in Cu oxidation state, and evolution of active sites are ubiquitous during extended CO_2_RR and can contribute to catalytic deactivation [79].

For organic layers maintained by weak interactions, potential changes and electrolyte composition can cause molecular reorientation, partial desorption, or interfacial migration. In [Bmim][PF_6_]-modified Cu, the IL is retained through interfacial ionic interactions, but cation and anion distributions remain responsive to the local electric field and ion-exchange processes. The [Bmim][PF_6_]-modified Cu catalyst maintained its catalytic performance over 40 h, as shown in Figure 7b [77]. XPS and elemental analyses separately support the retention and chemical evolution of the ionic-liquid-derived layer during electrolysis. Strongly anchored organic layers are more resistant to desorption but remain coupled to reconstruction of the Cu substrate. In a PIL-modified Cu^0^/Cu^+^ tandem catalyst, in situ Raman spectroscopy showed rapid reduction of surface Cu_2_O to Cu^0^ and formation of a dynamic Cu–PIL interface (Figure 7c), demonstrating that the organic network can persist even as its surrounding metal environment changes with potential [78]. During prolonged operation, organic layers may also undergo functional-group loss, ion exchange, or structural degradation. After the 40 h test, the PIL framework retained characteristic N 1s and C 1s signals, whereas the Cl 2p signal decreased markedly because of exchange between Cl^−^ and OH^−^. This result shows that the organic interface is not invariant, its main framework persists while local chemical evolution occurs. Post-test XRD and SEM further indicated that the catalyst crystal structure and morphology were largely preserved, supporting the stabilizing effect of the PIL network [78].

The organic–Cu interfacial stability should therefore be assessed from the perspective of dynamic evolution. Beyond molecular binding strength, surface reconstruction of Cu, electrolyte-ion interactions, and intermediate coverage must be considered together. Future interfaces should combine resistance to degradation with controlled structural responsiveness, allowing self-adaptation during long-term CO_2_RR at high current density.

#### 3.3.3. Strategies for Enhancing Interfacial Stability

To mitigate the desorption, structural rearrangement, and functional decay of organic interfaces during prolonged electrolysis, electrochemical oligomerization offers an effective strategy for enhancing molecular retention. In this approach, reduction of an N-arylpyridinium precursor forms a persistent oligomeric film on Cu, which suppresses molecular loss and stabilizes the organic–Cu interface under electrochemical conditions [80]. Stronger interfacial attachment can reduce molecular migration caused by electrolyte flow and electric fields. Covalent bonding, metal–ligand coordination, and multipoint interactions increase the degree of molecular fixation and improve long-term retention. In aryl-derived systems, controlled electrochemical deposition of a persistent organic film provides one route to reduced molecular loss, although a direct Cu–C covalent bond should not be assumed without specific evidence [80]. ODT forms a durable layer through Cu–S anchoring and intermolecular alkyl-chain interactions and maintains ethylene production after 10 h at 400 mA cm^−2^ [81]. Fourier-transformed EXAFS spectra show that the functionalized Cu nanowires remain dominated by Cu–Cu coordination, while weaker Cu–S, Cu–N, and Cu–O contributions are present in the modified surface region [81].

Continuous networks formed through polymer cross-linking, two-dimensional organic frameworks, or PIL coatings can restrict excessive migration of organic units and preserve interfacial integrity. PILs combine ionic-liquid functionality with a polymer backbone that immobilizes the ionic units and produces a stable organic network on Cu. In the PIL-modified Cu^0^–Cu^I^ tandem catalyst, N 1s and C 1s signals from the polymer backbone remained detectable after a 40 h CO_2_RR test, while XRD and SEM showed that the crystal structure. Post-electrolysis XPS, XRD, and SEM analyses indicated that the PIL-containing framework and the catalyst morphology were largely retained after prolonged operation [78]. Figure 8a illustrates the construction of a core–shell Cu@PIL interface through polymerization and cross-linking of ionic-liquid monomers around Cu nanoparticles [72].

Control over polymer architecture and interfacial organization can further balance stability with activity. Separately, Figure 8b presents the 40 h electrolysis performance of a PIL-based Cu^0^–Cu^I^ tandem catalyst at 150 mA cm^−2^, demonstrating the operational durability of a continuous ionic-polymer network [78]. Stabilization, however, should not be equated with simply maximizing binding strength. An overly rigid interface can lose its ability to respond to the reaction environment, whereas a flexible network that integrates noncovalent interactions can maintain integrity while retaining dynamic adaptability. Future organic–Cu interfaces should therefore balance molecular fixation, structural stability, and responsive reorganization for prolonged high-current-density CO_2_RR [72,78].

In summary, organic modification provides diverse strategies for constructing functional Cu-based CO_2_RR interfaces. Chemical bonding, noncovalent adsorption, polymer coatings, and ionic-liquid layers enable control over interfacial structure, stability, and dynamic behavior. The attachment mode, molecular geometry, and assembly architecture collectively determine retention under electrochemical conditions, while stabilization strategies preserve interfacial function during prolonged operation. However, the core value of organic molecular modification lies not only in creating stable interfaces but also in its ability to further modulate the electronic state, local microenvironment, and behavior of key reaction intermediates on the Cu surface. The following section therefore examines the catalytic mechanisms by which electronic effects, adsorption regulation, and microenvironment engineering alter CO_2_RR pathways and C_2+_ selectivity on Cu.

## 4. Interfacial Mechanisms by Which Organic Modifiers Enhance CO_2_RR Performance on Cu-Based Catalysts

Cu-based catalysts are among the most promising metal systems for producing C_2+_ products because they can efficiently promote C–C coupling during the electrochemical CO_2_RR. Nevertheless, bare Cu remains limited by insufficient product selectivity, competition from HER, and inadequate long-term stability. In recent years, organic molecular layers introduced onto Cu have enabled regulation of metal–molecule interactions without substantially altering the bulk Cu structure, thereby affecting the electronic state of the catalytic interface, the local reaction environment, and the conversion of key intermediates.

Building on the organic–Cu interface-construction strategies discussed in Section 3, the effects of organic modification on CO_2_RR can be organized into three principal categories. First, electronic coupling between organic molecules and Cu alters the electronic structure and adsorption properties of active sites. Second, organic interfacial layers regulate the local electrode/electrolyte environment, including wettability, ion distribution, and solvation structure. Third, molecules bearing specific functional groups can participate in key reaction steps through secondary-coordination-sphere effects or cooperative catalysis. This section therefore examines electronic-structure regulation, interfacial microenvironment control, and molecule–Cu cooperative catalysis as the major mechanisms through which organic modifiers enhance Cu-based CO_2_RR.

### 4.1. Electronic-Structure Regulation Induced by Molecular–Cu Interfaces

Interfacial coupling between an organic molecule and a Cu surface can alter the electronic structure of Cu active sites through charge transfer, orbital interactions, and local electron redistribution, thereby changing the adsorption of reactants and key intermediates. Compared with conventional metal doping or alloying, organic modification offers a highly designable molecular structure and enables finer control over the Cu surface electronic environment. Molecule-induced electronic regulation is therefore an important basis for the influence of organic–Cu interfaces on CO_2_RR selectivity [16,82,83].

#### 4.1.1. Regulation of Surface Charge and Local Electronic States

Organic molecules coupled to Cu can alter active-site electronic structure through molecule-induced charge transfer, orbital hybridization, and local electron redistribution. Electron-donating and electron-withdrawing functional groups influence the surface electron density through Cu–C, Cu–S, Cu–N, or noncovalent interactions and can consequently tune local electronic states and, in suitable systems, the position of the Cu d-band center. Unlike alloying or defect engineering, molecular modification provides a highly programmable design space while largely preserving the bulk Cu lattice. EXAFS measurements further show that modified catalysts remain dominated by Cu–Cu coordination, with Cu–S, Cu–N, or Cu–O coordination appearing primarily in the surface region [48]. This ligand-effect concept originates from homogeneous catalysis, where ligands regulate the electronic properties and reaction pathways of a metal center, and has increasingly been introduced into heterogeneous electrocatalytic interfaces. Electronic coupling between an organic molecule and the metal surface can alter the binding strength and adsorption configuration of carbon-containing intermediates. In particular, the coverage and binding mode of *CO, the central intermediate connecting CO_2_ reduction to C_2+_ formation, directly affect subsequent C–C coupling. By tuning Cu–intermediate electronic interactions, organic interfaces can redirect reaction pathways and improve C_2+_ selectivity [84].

Experimental studies provide direct evidence for molecule-induced regulation of Cu electronic structure. Long-chain alkyl modifiers bearing different terminal groups have been introduced onto Cu nanowires to compare their effects on the local electronic state and CO_2_RR performance. Octadecanethiol (ODT)-modified Cu nanowires showed enhanced ethylene production at high current density. At 400 mA cm^−2^, the ethylene Faradaic efficiency increased from approximately 30% on unmodified Cu nanowires to 53%, while HER was markedly suppressed and stable performance was retained for 10 h [81].The thiol headgroup formed Cu–S interactions and induced interfacial electron redistribution. Cu LMM Auger spectra reveal terminal-group-dependent Cu^0^/Cu^+^ surface-state distributions, with the Cu–SH system showing the most pronounced mixed-valence character. Separate Cu 2p XPS measurements also show binding-energy shifts after molecular modification [81]. Figure 9 shows that different terminal groups induce distinct degrees of interfacial charge redistribution at the Cu surface. The Cu-SH interface exhibits stronger charge coupling with *CO and preferentially stabilizes a top-site CO adsorption configuration [81]. Correspondingly, the barrier for *CO-*CO coupling was lower on Cu-SH than on Cu-NH and Cu-OH, favoring *OCCO formation and ethylene production. These results demonstrate that organic molecules can regulate Cu active-site properties through metal–molecule electronic interactions rather than serving solely as passive surface coverings [81].

N-containing organic molecules can similarly induce pronounced electronic regulation through their interaction with Cu. The lone pair on an N-heterocycle or pyridinium-derived species can perturb the electron distribution around surface Cu sites. Han and co-workers showed that N-substituted pyridinium modifiers on polycrystalline Cu substantially increased C_2+_ selectivity. N-phenylpyridinium and N-biphenylpyridinium derivatives produced high C_2_/C_3_ selectivity and strongly altered competition between C_2+_ and methane formation, indicating that N-containing organic layers can regulate the Cu interfacial environment and the conversion of carbon-containing intermediates [34]. Li and co-workers further achieved precise control over Cu–CO adsorption using arylpyridinium-derived films. At −0.83 V vs. RHE, the ethylene Faradaic efficiency reached 72% and total C_2+_ selectivity exceeded 75%, both substantially higher than those of bare Cu. In situ Raman spectroscopy and DFT calculations showed that the film increased the CO_atop_/CO_bridge_ ratio, whereas SEM and XRD confirmed that the bulk Cu morphology remained largely unchanged. The enhancement was therefore attributed to modulation of intermediate adsorption and interfacial electronic interactions rather than to reconstruction of the Cu bulk. Further analysis revealed a volcano-type relationship between ethylene selectivity and the Bader charge of the molecular N site, suggesting that an intermediate electron-donating capability optimizes CO adsorption and promotes C–C coupling [80].

In addition to Cu–S electronic coupling generated by organic thiols, adsorbed sulfur species can directly modify the electronic properties of Cu and redirect CO_2_RR pathways. Liang and co-workers found that S-doped Cu_2_O underwent in situ reconstruction during CO_2_RR to form metallic Cu sites decorated with adsorbed S. Charge-density-difference and projected-density-of-states analyses showed that S adsorption induces local charge redistribution at the Cu–S interface and modifies the orbital interactions between surface Cu/S sites and adsorbed intermediates. This altered electronic environment preferentially favors the formation and stabilization of the O-bound *OCHO intermediate, while making the adsorption of *H and *COOH less favorable. Consequently, the *OCHO-mediated pathway toward formate is promoted, whereas the competing hydrogen evolution reaction and the *COOH-mediated CO pathway are suppressed, resulting in a pronounced shift in selectivity toward formate [85]. Similar sulfur-mediated regulation of intermediate binding and preferential formation of *OCHO has also been reported for other S-modified Cu catalysts [64,86]. These findings demonstrate that Cu–S interfaces can redirect CO_2_RR selectivity by electronically differentiating the adsorption energetics of competing intermediates.

The molecule-induced electronic effects generally coexist with changes in wettability, interfacial electric fields, and local solvation, making their individual contributions difficult to isolate. Moreover, descriptors such as the d-band center are useful for rationalizing adsorption-energy trends but have limitations in describing transient structures at dynamic electrochemical interfaces. Future studies should combine operando XAS, FTIR, in situ Raman spectroscopy, and multiscale modeling to resolve the dynamic electronic structure of organic–Cu interfaces and establish more accurate relationships among molecular structure, interfacial electronic state, and catalytic performance [13,87].

#### 4.1.2. Dynamic Regulation of Cu Oxidation States and Interfacial Stabilization

The oxidation state of a Cu surface is not static during CO_2_ electroreduction but evolves dynamically in response to potential, electrolyte composition, and adsorbed intermediates. Operando studies suggest that Cu^+^ species can influence the surface electronic structure and the adsorption of carbon-containing intermediates, and dynamically formed Cu^+^/Cu^0^ interfaces are often considered more favorable for C_2+_ formation than a single oxidation state [50]. Whether Cu^+^ acts as an independent active center remains controversial, however, and an increasing body of evidence instead emphasizes a dynamically maintained mixed-valence interface [84].

The organic modifiers provide an effective means of influencing Cu oxidation-state evolution. Through coordination and interfacial electronic coupling, molecules containing N- or O-donor groups can modify the local coordination environment and slow the cathodic reduction of Cu oxides. In the polydopamine (PDA)-modified Cu_x_O system, the PDA coating altered the initial Cu valence distribution and formed an approximately nanometer-scale organic shell. More importantly, ex situ XPS, XRD, and electron microscopy after CO_2_RR showed that PDA substantially retarded the reduction of Cu^2+^ to Cu^+^/Cu^0^, leaving a dynamically mixed Cu^2+^/Cu^+^/Cu^0^ environment rather than allowing rapid complete metallization. At −1.4 V vs. RHE in a neutral electrolyte, Cu_x_O@PDA achieved an ethylene Faradaic efficiency of 53.4%, compared with approximately 31.1% for unmodified Cu_x_O, and displayed improved operational stability. XPS and structural characterization indicate that PDA retards the reduction of Cu oxide and helps preserve a dynamically mixed Cu^2+^/Cu^+^/Cu^0^ environment [88].

The polymeric organic layers can also improve the operational stability of Cu-based catalytic interfaces during prolonged electrolysis. Chen and co-workers introduced a polyvinylidene chloride (PVDC) coating onto CuO electrodes to regulate interfacial hydrophobicity and proton transport. With an optimized PVDC loading of 50 μg cm^−2^, the CuO–PVDC electrode achieved an ethylene Faradaic efficiency of 41.4% at −0.89 V vs. RHE, compared with 32.2% for bare CuO. During the 6 h stability test, both the C_2_H_4_ Faradaic efficiency and partial current density gradually decreased; however, the PVDC-modified electrode consistently exhibited better retention than bare CuO (Figure 10). In particular, the C_2_H_4_ partial current density decreased from approximately 6.8 to 5.0 mA cm^−2^ for CuO-PVDC, whereas that of bare CuO declined from approximately 5.8 to 3.2 mA cm^−2^. Although PVDC did not prevent the morphology evolution of CuO during electrolysis, it acted as a binder and helped maintain interfacial hydrophobicity, thereby mitigating the loss of catalytic activity. These results demonstrate that polymer coatings can enhance the durability of Cu-based interfaces through microenvironment regulation and improved catalyst retention [89].

The oxygen-coordinating molecules bearing carboxylate or phosphonate groups may likewise influence Cu oxidation-state evolution through O–Cu interactions. Nevertheless, the optimal fraction of Cu^+^ and its exact role in CO_2_RR depend strongly on catalyst structure, electrolyte composition, and operating conditions. The prevailing view is that Cu^+^ functions as part of a dynamic mixed-valence interface rather than as an isolated, universally active center. Operando XAS, in situ infrared and Raman spectroscopy, and theoretical calculations are therefore needed to resolve the relationship between oxidation-state evolution and reaction pathways under organic modification.

#### 4.1.3. Optimization of Local Electronic Structure Through Coordination-Environment Engineering

Beyond interfacial charge transfer and oxidation-state regulation, organic molecules can alter the local chemical structure of Cu active centers by constructing defined coordination environments. Unlike elemental doping or alloying, N, O, and S heteroatoms in organic ligands use lone-pair electrons to interact with Cu, thereby tuning the first coordination shell, local electron distribution, and active-site geometry without substantially changing the bulk Cu structure. Coordination-environment engineering is therefore a powerful strategy for precisely controlling Cu-based CO_2_RR pathways [90]. For molecular coordination at metallic Cu surfaces, the ligand–Cu interaction primarily perturbs the local electronic environment of accessible surface Cu sites while retaining the extended metallic Cu structure. The coordination structure around a Cu active site directly influences adsorption and conversion of carbon-containing intermediates [90]. For example, Cu–N coordination can adjust the electronic state of Cu to provide a more appropriate binding strength for *CO, increase surface CO coverage, and facilitate C–C coupling. Rational selection of ligand identity and coordination geometry can thus tailor the local reaction environment of the Cu center [84].

Ligand-coordinated Cu nanoclusters represent a distinct class because their coordination environment forms an intrinsic part of the active-site structure rather than merely perturbing an extended metallic Cu surface. XANES and EXAFS measurements show that varying the N/Cu ratio changes the local coordination environment of ultrafine Cu nanoclusters [91]. Using this strategy, N-coordinated Cu nanoclusters were prepared with markedly enhanced ethylene formation relative to unmodified Cu clusters. The optimized experimental catalyst, Cu–N(ii)/NC, achieved an ethylene Faradaic efficiency of 61.4% at −1.05 V vs. RHE and a current density of approximately −200 mA cm^−2^, whereas bare Cu nanoparticles showed less than 5% ethylene selectivity. DFT models containing different numbers of coordinated N atoms clarified the electronic origin of this behavior. The barrier for CO-CO coupling decreased from 1.28 eV on the CuN_1_/NC model to 0.35 eV on CuN_8_/NC. Increasing N coordination progressively shifted the d-band center downward and weakened CO adsorption; the CuN_8_/NC model provided a favorable combination of moderate CO adsorption and a low coupling barrier [91].

Coordination polymers constitute another distinct class in which Cu centers are structurally integrated into an extended ligand environment, resulting in site accessibility and local coordination structures different from those of molecularly modified metallic Cu surfaces and ligand-coordinated nanoclusters. In Cu coordination polymers constructed from the same organic linker, changing the Cu precursor and synthesis conditions generated either Cu–N_2_S_2_ or Cu–I_2_S_2_ local coordination motifs, leading to markedly different CO_2_RR selectivity [68]. In situ ATR-FTIR spectra showed that Cu–N_2_S_2_ exhibited atop- and bridge-bound CO signals of comparable intensity during reaction, whereas Cu–I_2_S_2_ displayed a markedly unbalanced adsorption distribution [68]. Consistent with this difference, Cu–N_2_S_2_ achieved Faradaic efficiencies of 61.2% for ethylene and 82.2% for total C_2+_ products. Calculations showed that N/S coordination shifted Cu 3d states toward the Fermi level, stabilized *OCCO, and lowered energetic barriers along the C_2+_ pathway, while Cu–I_2_S_2_ favored *OCHO and formate formation [68]. Organic framework materials such as covalent organic frameworks (COFs) and metal–organic frameworks (MOFs) further extend this type of structurally integrated coordination control to ordered and spatially confined environments. Site-defined N donors in these frameworks can stabilize uniform Cu–N_x_ centers, suppress Cu migration and aggregation, and tune intermediate adsorption. Thus, an organic framework serves not only as a support but also as an active coordination environment that regulates the electronic properties and reaction pathways of Cu centers [92].

Coordination-environment engineering is generally accompanied by changes in electronic structure, local electric fields, and solvation, and the independent contribution of each factor remains difficult to separate. Although molecularly coordinated Cu surfaces, ligand-coordinated Cu nanoclusters, and coordination polymers or framework-confined Cu sites all involve Cu-ligand interactions, they should not be regarded as mechanistically equivalent. Molecular coordination mainly perturbs accessible metallic Cu surface sites, whereas nanoclusters possess more strongly coordination-defined active structures, and Cu centers in coordination polymers or organic frameworks are embedded within extended ligand environments with different geometric constraints and site accessibility. Future work should combine operando XAS, in situ infrared and Raman spectroscopy, and theoretical simulations to track Cu–ligand evolution under working conditions and establish clearer structure–property relationships among ligand structure, coordination environment, and CO_2_RR performance [13].

Overall, electronic regulation by organic interfaces should not be interpreted in terms of a single descriptor. Changes in surface charge, Cu oxidation state, and local coordination environment are often strongly coupled and may also evolve dynamically under CO_2_RR conditions. Therefore, correlations between C_2+_ selectivity and parameters such as the d-band center, Cu^+^ fraction, or coordination number should be interpreted cautiously unless the active interfacial structure is verified under operando conditions.

### 4.2. Regulation of the Local Microenvironment by Organic Interfaces

In addition to tuning the electronic structure of Cu, organic modifiers influence CO_2_RR by regulating the local microenvironment at the electrode/electrolyte interface. Under reaction conditions, local CO_2_ enrichment, solvation structure, ion distribution, and proton-transfer behavior all affect the formation and conversion of key intermediates. Because their molecular structures and functional groups are programmable, organic layers can modify interfacial wettability, double-layer organization, and local electric fields while leaving the bulk Cu framework largely unchanged. Microenvironment regulation therefore represents another major mechanism by which organic-modified Cu catalysts achieve improved CO_2_RR selectivity.

#### 4.2.1. Hydrophobic Interfaces for Regulating Reactant Transport and Competition with HER

Cu electrodes in aqueous electrolytes generally develop a highly hydrated interfacial layer. Excessive hydration can intensify water-mediated side reactions and reduce the effective concentration of CO_2_ and carbon-containing intermediates at the catalytic interface. Surface water structure and local solvation have an important influence on competition between CO_2_RR and HER, and redistribution of interfacial water can alter both reactant transport and proton participation [93]. Constructing a moderately hydrophobic organic layer can therefore reorganize water near the electrode, improve CO_2_ transport, and shift the balance between CO_2_RR and HER. Hydrophobic regulation should not be understood as simply excluding water; rather, it must optimize mass transfer at the gas–liquid–solid triple-phase boundary while retaining the proton supply required for CO_2_RR. An appropriate degree of hydrophobicity is therefore essential for maximizing C_2+_ selectivity [76].

Wakerley and co-workers constructed a bio-inspired hydrophobic Cu interface by modifying the surface with a long-chain organic layer. The water contact angle increased from approximately 17° to 153°, producing a stable gas–liquid–solid triple-phase interface [76]. In 0.1 M CsHCO_3_, the ethylene Faradaic efficiency increased from 9% on unmodified Cu to 56%, the ethanol Faradaic efficiency increased from 4% to 17%, and the total C_2+_ Faradaic efficiency reached 65% at a current density of approximately 260 mA cm^−2^, while the HER Faradaic efficiency decreased from 71% to 10%. After 12 h at 15 mA cm^−2^, the contact angle remained 143°. These results show that a hydrophobic organic layer can increase local CO_2_ availability, suppress water-driven HER, and optimize transport at the triple-phase boundary [76].

Hydrophobic microenvironments can also be generated through the intrinsic structure of a Cu catalyst rather than through molecular or polymer modification. Niu and co-workers developed a hierarchical, intrinsically hydrophobic Cu gas-diffusion electrode. Its high-curvature dendritic architecture stabilized the gas–liquid–solid interface and mitigated electrolyte flooding at high current density. In a flow cell, the electrode delivered a C_2+_ partial current density of 255 mA cm^−2^ with a C_2+_ Faradaic efficiency of approximately 64% and maintained stable operation for 45 h at 300 mA cm^−2^. The morphology-derived hydrophobicity created a robust triple-phase interface without masking active sites, thereby sustaining CO_2_ transport and suppressing side reactions (Figure 11a) [94]. Although this example does not involve an organic modifier, it illustrates the general mass-transport principles that hydrophobic organic interfaces seek to reproduce. Liang and co-workers similarly modified Cu with hydrophobic polymers such as poly (vinylidene fluoride) (PVDF) and polyethylene (PE). The coatings restricted interfacial water diffusion, suppressed HER, and increased the local pH from 9.2 to 9.4, thereby improving CO retention and the environment for C–C coupling. In an H-type cell at −1.0 V vs. RHE, the ethylene Faradaic efficiency increased from 16.7% on the unmodified CuO-derived electrode to 32% after PVDF modification and to 40% after PE coating, with modified electrodes outperforming bare Cu across the investigated potential window. The calculated OH^−^ concentration profiles indicate that hydrophobic polymer coatings alter interfacial water transport and the local acid–base environment, thereby creating conditions more favorable for CO retention and C-C coupling [95]. Fluorinated polymer coatings provide another route to a low-surface-energy organic interface. Zhao and co-workers coated Cu nanoparticles in situ with a fluorinated polymer, decreasing interfacial water wettability, increasing CO coverage, and altering interfacial polarity and intermediate stabilization. In a membrane-electrode-assembly (MEA) electrolyzer using a bicarbonate electrolyte at a cell voltage of −3.98 V, the C_2+_ Faradaic efficiency of the Cu-poly catalyst increased from 61.59% on bare Cu to 71.08%, corresponding to a C_2+_ partial current density of 355.4 mA cm^−2^. Ethylene selectivity increased concurrently with the overall C_2+_ Faradaic efficiency, supporting the role of the hydrophobic organic interface in suppressing HER and promoting C–C coupling [96].

Overall, the function of a hydrophobic organic interface extends beyond increasing the macroscopic contact angle. Such interfaces coordinately regulate CO_2_ transport, interfacial water organization, and proton delivery, thereby optimizing competition between CO_2_RR and HER. Future designs should balance gas enrichment, ion transport, and reactant accessibility rather than simply maximizing hydrophobicity.

#### 4.2.2. Regulation of Proton Transfer and the Local Acid–Base Environment

CO_2_ electroreduction involves multiple proton-coupled electron-transfer (PCET) steps; consequently, the interfacial proton-supply rate, solvation structure, and local acid–base environment strongly influence kinetics and product distribution [2]. Unlike regulation through the bulk electrolyte alone, an organic layer can actively control proton accessibility and local pH through its functional groups, ionic conductivity, and spatial organization, thereby affecting key-intermediate formation and competition between CO_2_RR and HER. Potential-dependent changes in the interfacial bicarbonate-related spectral intensity at different Nafion loadings indicate that the ionomer modifies the local acid–base environment and the distribution of interfacial species [73].

Ionomer modification is an important strategy for controlling interfacial proton transport. Polymers such as Nafion contain sulfonic acid groups that mediate ion exchange, while their hydrophobic backbones limit excessive water coverage and improve CO_2_ transport. On Cu, Nafion altered interfacial water structure and proton-transfer behavior and substantially improved C_2_ selectivity. At −1.2 V vs. RHE, an optimally loaded Cu@Nafion electrode increased the C_2_ Faradaic efficiency from 28.3% on bare Cu to 73.5% and decreased the HER Faradaic efficiency from 40.6% to 16.8%. These results demonstrate that moderate ionomer coverage suppresses competitive proton reduction while promoting C_2+_ formation [73]. Poly(ionic liquid)s (PILs) have also been used to construct organic interfaces with tailored ion-transport properties [78]. By covalently fixing ionic-liquid units to a polymer backbone, a PIL reduces migration of the active organic component while retaining ionic conductivity and interfacial regulation. At a Cu electrode, the PIL layer can influence ion migration and local acid–base chemistry, optimize proton transfer, and suppress HER under high-current-density conditions. For example, the Cu^0^–Cu^I^@PIL tandem catalyst achieved a C_2+_ Faradaic efficiency of 76.1% at −0.85 V vs. RHE and maintained appreciable C_2+_ selectivity at high current density, demonstrating that a stable ionic interface can preserve a favorable reaction microenvironment. An appropriate ionomer loading can improve proton supply and the local reaction environment, whereas an excessively thick polymer layer can impede CO_2_ diffusion and reduce active-site accessibility. Ionomer design must therefore balance proton transport, CO_2_ delivery, and exposure of catalytic sites [97].

Acid–base-responsive organic molecules provide an additional means of regulating local proton concentration and CO_2_RR pathways. Amine-containing alkaline ionic liquids (AILs), for example, undergo reversible protonation and deprotonation, increase the local interfacial pH, decrease the probability of H^+^ reduction to H_2_, and stabilize CO_2_RR intermediates. Cu@AIL retained high C_2+_ selectivity at very high reaction rates. In a flow cell, it achieved a C_2+_ Faradaic efficiency of 81.4% at −0.76 V vs. RHE and 0.9 A cm^−2^; even at 1.8 A cm^−2^ [98]. The calculated free-energy profiles in Figure 11b provide mechanistic support that the alkaline ionic-liquid microphase modifies the deep-reduction pathway relative to bare Cu [98].

Overall, regulation of proton transfer and local acid–base chemistry by organic interfaces is not equivalent to indiscriminately decreasing proton concentration. The objective is to retain the PCET steps required for CO_2_RR while suppressing excessive hydrogenation and HER. Future studies combining in situ spectroscopy, electrochemical kinetic analysis, and multiscale simulation are needed to clarify the relationships among water networks, ion migration, and CO_2_RR selectivity within organic interfaces.

**Figure 11:**
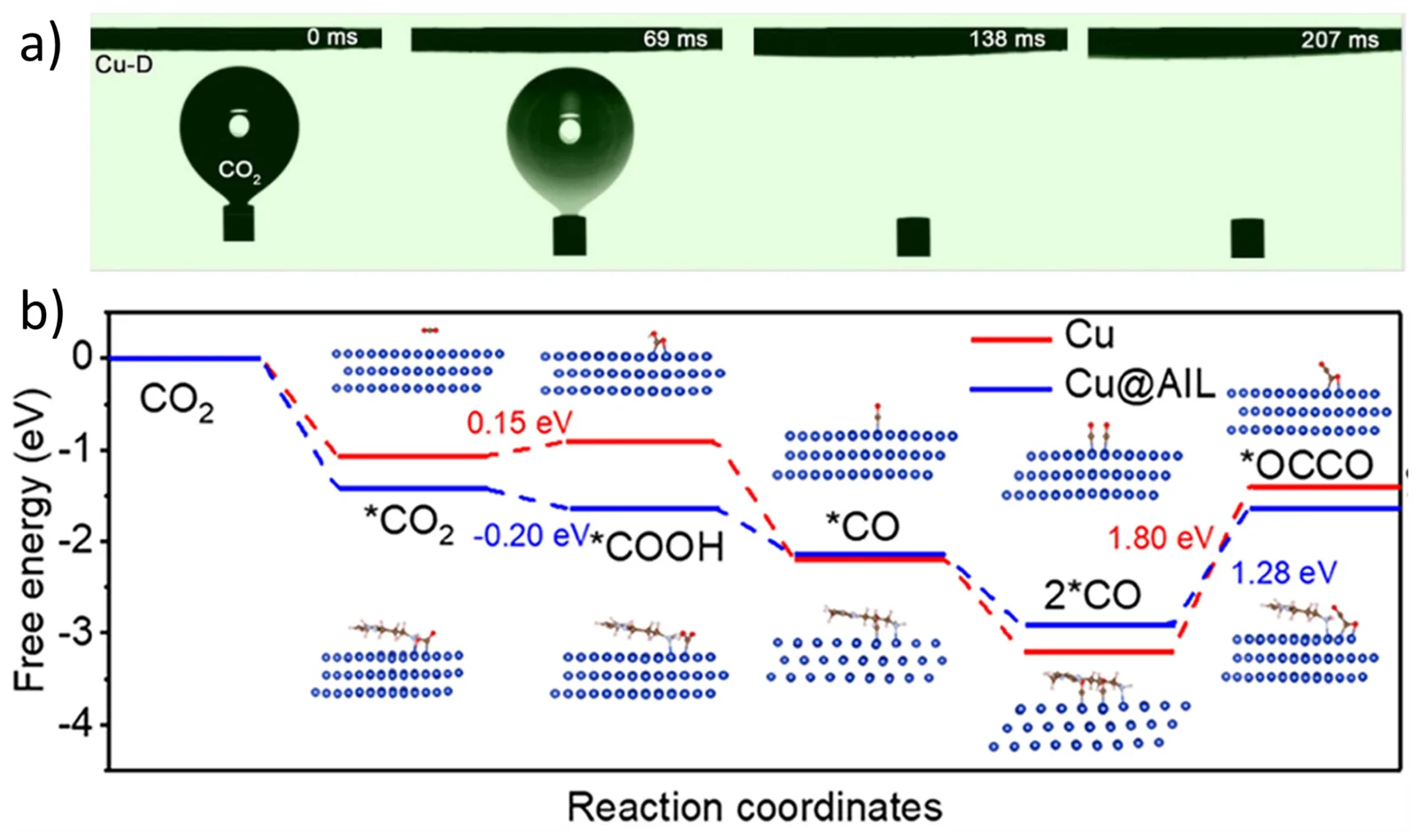
Regulation of reactant transport, interfacial water, and the local acid–base environment by organic interfaces. (**a**) Time-resolved droplet-wetting behavior on a hierarchical, intrinsically hydrophobic Cu gas-diffusion electrode. Reproduced under terms of the CC BY-NC-ND 4.0 license [94]. Copyright 2021, American Chemical Society. (**b**) Calculated free-energy profiles for CO_2_ reduction on bare Cu and alkaline-ionic-liquid-modified Cu. Reproduced with permission from Ref. [98]. Copyright 2023, American Chemical Society.

#### 4.2.3. CO_2_ Enrichment and Local-Concentration Effects

In addition to regulating proton transfer and interfacial water, organic layers can enrich CO_2_ near the catalytic interface and thereby increase the effective reactant concentration, alleviating mass-transfer limitations at high current density. Because CO_2_ has limited solubility in aqueous electrolytes, diffusion from the bulk phase to the catalyst can limit the CO_2_RR rate. Figure 12a illustrates how the near-surface CO_2_ concentration changes with increasing current density and highlights the more severe CO_2_ depletion associated with conventional H-cell transport relative to gas-diffusion-layer delivery [99]. Figure 12b further shows the coupled transport of CO_2_ and OH^−^ within a gas-diffusion-electrode catalyst layer, where the CO_2_ diffusion pathway is shortened to approximately 50 nm [99]. Organic modifiers containing CO_2_-philic functional groups may complement these electrode-level transport strategies by increasing local CO_2_ accessibility within the catalyst layer. [100,101].

The polar functional groups, amines, and ionic groups can strengthen interactions between CO_2_ and the interface through dipolar, electrostatic, or reversible-binding interactions, thereby increasing the local CO_2_ concentration. Amine-functional materials, for example, reversibly bind CO_2_ and can improve both capture and transport. Such CO_2_-affinitive groups are increasingly incorporated into electrocatalytic interfaces to improve gas supply within the catalyst layer and alleviate reactant-transport limitations at high current density [101]. Microporous organic materials can also improve interfacial CO_2_ transport by modifying gas-diffusion pathways. Polymers of intrinsic microporosity (PIMs) possess high free volume and tunable pores and can therefore serve as CO_2_-enriching interfacial materials. Nevertheless, enrichment addresses reactant supply rather than the intrinsic requirement for appropriate CO adsorption and C–C coupling at Cu active sites [100]. Ionic liquids and PILs are particularly attractive because of their high CO_2_ solubility and tunable ionic structures. Imidazolium-based ILs can enhance interfacial dissolution and diffusion of CO_2_ and modify the local solvation environment, improving transport to catalytic sites. Fixing ionic-liquid units on a polymer backbone retains CO_2_ affinity while improving stability. At −0.85 V vs. RHE, the poly (ionic liquid)-based Cu^0^–Cu^I^ tandem catalyst achieved a C_2+_ Faradaic efficiency of 76.1% and a C_2+_ partial current density of 304.2 mA cm^−2^, as shown in Figure 12c. Even at −1.25 V and a total current density of approximately 1000 mA cm^−2^, the C_2+_ Faradaic efficiency remained 46.0% [78].

More generally, gas-diffusion electrodes and microporous layers shorten CO_2_ transport pathways and increase gas accessibility within the catalyst layer. PIM-based interfaces are conceptually attractive because of their high free volume and adjustable pore architecture, although CO_2_ enrichment alone cannot determine C_2+_ selectivity; the adsorption of CO on Cu and the subsequent C–C coupling steps remain decisive [99].

Organic interfaces therefore regulate local CO_2_ concentration not merely by increasing the amount adsorbed but by optimizing dissolution, diffusion, and interfacial availability. At the high current densities required for practical CO_2_RR, achieving a coordinated balance among CO_2_ enrichment, ion transport, and active-site exposure will be a key objective of molecular interface design.

#### 4.2.4. Regulation of Interfacial Electric Fields and Water Structure

Organic layers influence CO_2_RR not only by changing surface chemistry but also by regulating charge distribution, molecular dipole orientation, and solvation at the electrode/electrolyte interface. The programmable charge and dipole characteristics of organic molecules can alter the Helmholtz layer and local electric field after adsorption on Cu, thereby affecting CO_2_ polarization, electron transfer, and stabilization of carbon-containing intermediates. Interfacial electric-field regulation is therefore an important mechanism of organic modification [84].

The charged organic molecules and ionic additives are representative tools for regulating interfacial electric fields. Thevenon and co-workers used the organic salt N, N′-ethylene-phenanthrolinium dibromide to modify polycrystalline Cu in CO_2_-saturated NaHCO_3_. The additive generated a persistent organic layer, changed the local reaction environment, and influenced intermediate coverage. The N, N′-ethylene-phenanthrolinium additive induced corrosion-driven formation of cube-like Cu nanostructures and generated a protective organic layer on the reconstructed Cu surface. The resulting catalyst reached a C_2+_ Faradaic efficiency of approximately 70% and maintained its selectivity for more than 40 h. Figure 12d schematically illustrates the additive-induced nanostructuring and subsequent stabilization of the Cu surface rather than directly displaying the catalytic performance data [102]. Surfactants can similarly reorganize the interfacial charge and wetting environment. Cetyltrimethylammonium bromide (CTAB) adsorbs through its quaternary-ammonium headgroup and changes surface wettability and double-layer organization. In the cited study, CTAB modification increased the Faradaic efficiency for formate from approximately 40% to approximately 71% and improved stability [103].

The imidazolium-based ionic liquids likewise affect CO_2_RR through interfacial charge distribution and solvation. The stable cationic center and tunable molecular structure of an imidazolium ion influence ion organization, polarity, and the local electric field near an electrode. Mechanistic studies—often using Ag or other model electrodes—show that imidazolium ions can stabilize polarized CO_2_-derived intermediates, reorganize the double layer, and modify interfacial electron transfer [104]. These general principles provide a basis for understanding ionic organic layers on Cu, but evidence obtained on non-Cu model surfaces should not be presented as direct structural proof for Cu. Organic modifiers also influence interfacial water. Because water participates in proton transfer and defines the solvation environment, its orientation, hydrogen-bond network, and local organization affect reaction kinetics. Hydroxyl, amino, carboxylate, and N-heterocyclic groups can reorganize interfacial water through hydrogen-bonding and dipolar interactions, thereby modifying solvation and PCET [93].

The interfacial electric fields, water structure, and ion distribution are strongly coupled, and their independent contributions remain difficult to separate. Adsorption of an ionic liquid or charged molecule changes not only the interfacial charge distribution but also water orientation and ion solvation. Future studies should combine in situ infrared spectroscopy, surface-enhanced Raman spectroscopy, X-ray absorption spectroscopy, and molecular-dynamics simulations to establish quantitative relationships between organic-interface structure and the local electrochemical environment [93].

Although regulation of hydrophobicity, local pH, CO_2_ availability, and interfacial electric fields can improve CO_2_RR selectivity, these effects are not necessarily maximized simultaneously. For example, excessive hydrophobicity may restrict water and proton accessibility, whereas excessive proton availability can enhance HER or promote competing hydrogenation pathways. Therefore, the key challenge is to establish an optimal interfacial microenvironment rather than simply maximizing a single parameter.

### 4.3. Cooperative Catalytic Mechanisms Between Organic Molecules and Cu Active Sites

Beyond electronic-structure regulation and local-microenvironment control, some organic layers form spatially coupled catalytic interfaces with Cu active sites and directly influence key elementary steps of CO_2_RR. These molecules commonly contain pyridyl or imidazolyl N atoms, amine groups, or O-donor functionalities and can act as a secondary coordination sphere. By regulating the local coordination environment, reactant orientation, and proton-transfer pathways around Cu sites, they alter the catalytic reaction network [84].

Unlike homogeneous molecular catalysts, in which a metal center and its ligands jointly complete a molecular catalytic cycle, organic molecules in heterogeneous Cu systems generally do not function as independent active centers. Instead, they cooperate with the Cu surface to regulate reaction kinetics. Accordingly, the field is shifting from the simple organic modification toward the deliberate cooperative design of molecular functional units and metal active sites.

#### 4.3.1. Cooperative CO_2_ Activation by Organic Molecules and Cu Active Sites

The initial activation of CO_2_ involves conversion of linear CO_2_ into a bent, polarized *CO_2_-configuration. This high-energy transformation is commonly regarded as a kinetically limiting step in CO_2_RR. Constructing an interfacial environment that stabilizes the polarized state is therefore an important strategy for lowering the activation barrier [2].

Rather than relying solely on direct adsorption of CO_2_ by Cu, a functional organic molecule can create a secondary coordination environment that provides additional electronic and steric regulation near the active site. N-containing groups with lone-pair electrons can influence CO_2_ adsorption through Cu–N coordination, dipolar interactions, or local electric-field effects, promoting bending and initial electron transfer [84]. Imidazolium and pyridinium species are particularly useful because they combine a stable charge center with tunable molecular structure. The landmark study of EMIM-BF_4_ used Ag rather than Cu as the model electrode, but it established a broadly relevant mechanistic principle, an imidazolium cation can stabilize a high-energy CO_2_-derived intermediate and lower the barrier for the first electron-transfer step (Figure 13) [29]. In Cu systems, analogous charged organic species are therefore best understood as components of a secondary coordination sphere that regulate the electronic and spatial environment around Cu, rather than as independent catalytic centers.

Molecular modifiers can also promote cooperation between a Cu site and an organic functional unit through controlled spatial organization. Nam and co-workers emphasized that organic layers are not merely passive coverings but can function as secondary coordination environments that influence CO_2_ activation, proton transfer, intermediate stabilization, and surface reaction pathways [84]. Phenanthrolinium, pyridinium, and related modifiers have produced substantial improvements in C_2+_ selectivity on Cu, illustrating the importance of cooperative organic–inorganic interfaces for accelerating CO_2_RR [84].

#### 4.3.2. Stabilization of Key Intermediates and Promotion of C–C Coupling by Organic Molecules

In pathways to C_2+_ products, the surface coverage of *CO, the coupling of CO-derived species, and subsequent C–C bond formation are decisive determinants of product selectivity. An organic layer can construct a secondary coordination environment around Cu sites and provide spatial confinement, noncovalent interactions, or local coordination that changes intermediate adsorption configurations and reaction barriers. Rather than acting only through the intrinsic electronic structure of Cu, such cooperative interfaces enhance C_2+_ selectivity by stabilizing high-energy intermediates and reorganizing reaction pathways [84,105].

Yao and co-workers reported that an in situ deposited polyaromatic layer persisted on Cu and promoted C_2+_ formation by modifying the local surface environment and the adsorption behavior of CO intermediates. The organic film was proposed to increase the effective local population and favorable arrangement of adjacent CO species, thereby facilitating subsequent C–C coupling without itself completing a catalytic cycle. The system therefore illustrates how an organic layer can function as a secondary coordination environment for regulating Cu-surface pathways [106]. Because the cited study primarily establishes interfacial-film effects and durability, claims of a specific DFT-demonstrated CO-adsorption geometry should be made only when supported by the corresponding original calculations.

Organic molecules containing specific functional groups can also enhance C_2_ selectivity by stabilizing intermediates. Chen and co-workers constructed a composite coating of benzotriazole (BTA) and poly (vinyl chloride) (PVC) on Cu, combining small-molecule interactions with polymer confinement. BTA was confined within the PVC matrix and stabilized *COOH, *COCOH, and *COCHO through hydrogen bonding, while PVC maintained the organic interface through van der Waals interactions and spatial confinement. In CO_2_-saturated 0.1 M KHCO_3_, the C_2_ Faradaic efficiency increased from approximately 36% on bare Cu to 71.0 ± 5.6%, with favorable long-term stability. The coating promoted C–C coupling by stabilizing key C_1_ and C_2_ intermediates rather than through a directly demonstrated Cu–N covalent coordination bond [107]. Ionic organic molecules can similarly create an adsorption environment favorable for the C_2_ pathway. He and co-workers modified a Cu gas-diffusion electrode with pyridinium-based ionic liquids and improved ethylene selectivity in a flow-cell system. Cu@[BPy]Br-2.4 achieved an ethylene Faradaic efficiency of 31.88% at −1.15 V vs. RHE and a current density of 275 mA cm^−2^, while hydrogen evolution was substantially suppressed [108]. Theoretical calculations further indicated that the pyridinium-based ionic-liquid interface induced interfacial electron redistribution and altered the energetics of CO_2_-derived intermediate formation and conversion. These interfacial effects modified the adsorption behavior of CO_2_ and carbon-containing intermediates and promoted the utilization of surface-bound CO, thereby creating more favorable conditions for C–C coupling [108].

Organic coordination structures with defined confinement can provide an additional level of cooperative intermediate regulation. An Ag-decorated Cu coordination polymer (Ag@Cu-DM) combined CO-generating Ag-related sites with a porous Cu–organic framework. At −0.73 V vs. RHE, the total Faradaic efficiency for hydrocarbon liquid products reached 89%. Ag sites were proposed to increase *CO formation and surface coverage, while the porous Cu coordination-polymer environment shortened diffusion distances for CO_2_ and carbon-containing intermediates, facilitating tandem conversion and subsequent C–C coupling [109]. This mechanism was inferred from experimental results and prior mechanistic knowledge; the study did not directly calculate a C–C coupling barrier.

Overall, the enhancement of C_2+_ formation by organic molecules does not arise from a simple coverage effect. Instead, a secondary coordination environment changes the adsorption state, spatial distribution, and conversion pathway of key intermediates. Stabilization of *CO, promotion of *OCCO formation, and reduction in C–C coupling barriers are central mechanisms through which cooperative organic–Cu interfaces improve C_2+_ selectivity.

#### 4.3.3. Cooperative Catalysis by Ionic Liquids and Poly(Ionic Liquid)s

Ionic liquids are important platforms for constructing cooperative organic–Cu interfaces because they combine high CO_2_ affinity, tunable ionic structures, and diverse interfacial interactions. Unlike conventional neutral organic layers, ionic liquids can regulate metal/electrolyte interfaces through the combined effects of their cationic and anionic components [105]. In an Ag-foil descriptor study, changing the imidazolium cation–anion combination substantially altered the relative Faradaic efficiencies for CO and H_2_, as shown in Figure 14a [110]. Because this dataset was obtained on Ag, it provides general mechanistic guidance for ionic-liquid-mediated interfacial regulation rather than direct evidence for an identical pathway on Cu. [105].

Imidazolium-based ionic liquids are among the most extensively investigated organic promoter systems. Cations such as EMIM^+^ and BMIM^+^ can modify the interfacial electric field, impose local spatial organization, and influence CO_2_ activation, intermediate adsorption, and subsequent product formation. Theoretical and spectroscopic studies generally support interfacial-field and secondary-coordination-sphere effects rather than a complete catalytic cycle centered on the organic cation. Although carbene formation has been proposed in some systems, both its formation conditions and its catalytic contribution remain debated (Figure 14d). The more broadly supported interpretation is that imidazolium ions form a dynamic organic interface that cooperatively regulates metal active sites and intermediate conversion [110].

The anion also plays an important role in ionic-liquid promotion. Different anions influence the acid–base equilibrium of the imidazolium cation, interfacial ionic organization, and the resulting availability of the catalytically promoting cationic species. Weak Lewis-basic anions suppress deprotonation of EMIM^+^ or BMIM^+^ to a carbene, whereas strongly Lewis-basic anions promote carbene formation. The carbene may occupy electrode sites or form a stable carboxylate with CO_2_, both of which can reduce CO_2_RR activity (Figure 14b) [110]. Because the descriptor study supporting this interpretation was performed on Ag-based model systems, its conclusions should be treated as general ionic-liquid mechanistic guidance rather than direct proof for Cu. When free ionic liquids are introduced as physically adsorbed or deposited interfacial modifiers, rather than covalently immobilized species, their interfacial distribution can evolve during prolonged electrolysis. Finite miscibility or solubility in the surrounding aqueous electrolyte can lead to gradual partitioning and leaching of ionic-liquid components into the bulk phase, while concentration gradients and the applied electric field can promote diffusion, ion migration, and potential-dependent reorganization of the interfacial ionic layer. In porous catalyst layers, changes in wetting and liquid distribution during continuous gas and electrolyte transport may additionally redistribute physically retained ionic liquid and alter its local coverage or pore-filling degree. The relative importance of these processes depends strongly on ionic-liquid hydrophobicity and molecular structure, catalyst porosity, ionic-liquid loading, electrode potential, and cell configuration; therefore, phase separation or capillary displacement should not be regarded as universal degradation mechanisms for all ionic-liquid-modified CO_2_RR systems [111,112]. PILs mitigate these limitations by covalently incorporating ionic-liquid functionalities into a polymer backbone, thereby restricting macroscopic migration while retaining the local ionic and coordination environment characteristic of ionic liquids [111,112,113].For example, a PIL-based Cu^0^–Cu^1^ tandem catalyst achieved a C_2+_ Faradaic efficiency of 76.1% at −0.85 V vs. RHE, corresponding to a C_2+_ partial current density of 304.2 mA cm^−2^. At −1.25 V and a total current density of approximately 1000 mA cm^−2^, the C_2_^+^ Faradaic efficiency remained 46.0%. During a 40 h test at 150 mA cm^−2^, the catalyst retained appreciable C_2+_ selectivity, although the electrode was cleaned and dried approximately every 10 h, as shown in Figure 8b [78]. HRTEM further reveals adjacent Cu^0^ and Cu_2_O domains confined within the PIL framework (Figure 14c), supporting the proposed Cu^0^–PIL–Cu^1^ tandem interfacial structure [78]. The PIL framework reduced migration of ionic-liquid components, stabilized Cu-containing interfacial domains, and created a persistent secondary coordination environment [78].

Ionic liquids and PILs therefore represent an advanced stage in the development of cooperative organic–Cu interfaces. Their function extends beyond passive surface modification, cations influence CO_2_ activation and intermediate conversion through interfacial charge and secondary-coordination-sphere effects; anions regulate ionic structure and cation speciation; and the polymer backbone enhances long-term retention. Nevertheless, unlike homogeneous molecular catalysts, most organic components in these systems have not been shown to independently complete an entire CO_2_RR catalytic cycle. Operando spectroscopy, isotope labeling, and multiscale theory are needed to identify their true roles under working conditions and to establish quantitative relationships among organic structure, interfacial configuration, reaction pathway, and catalytic performance.

Organic molecules can cooperate with Cu sites in CO_2_ activation, intermediate stabilization, and C–C coupling; however, direct identification of the corresponding catalytic ensembles remains challenging. In many cases, the proposed synergistic mechanisms are inferred from theoretical calculations or indirect spectroscopic evidence. Future studies should therefore combine operando characterization with potential-dependent simulations to determine the identity, lifetime, and structural evolution of the true organic–Cu active interface.

## 5. Summary and Future Perspectives

### 5.1. Summary

Organic modification provides a versatile route for regulating Cu-based CO_2_ electroreduction beyond conventional control of catalyst composition, morphology, and crystallographic structure. This review has examined organic-modified Cu catalysts from an integrated interface-engineering perspective, covering the construction, stability, and catalytic functions of organic–Cu interfaces. Depending on the dominant mode of interaction with Cu, these interfaces can be established through covalent bonding or coordination, reversible noncovalent adsorption, and continuous polymer- or ionic-liquid-derived layers. These construction strategies determine not only the initial attachment of the modifier but also its molecular organization, accessibility to reactants, and ability to remain functional under electrochemical operating conditions.

The effects of organic modification strategy on Cu-based catalysts for CO_2_RR can be understood through three aspects. First, molecule–Cu interactions can alter the local charge distribution, coordination environment, oxidation-state evolution, and adsorption properties of surface Cu sites. Second, organic layers can reconstruct the interfacial microenvironment by regulating CO_2_ and water accessibility, local pH, ion distribution, electric fields, solvation structures, and proton transport. Third, functional groups and spatially organized organic domains can cooperate with Cu active sites to stabilize CO_2_-derived intermediates, adjust the relative populations of atop- and bridge-bound *CO, facilitate C–C coupling, and redirect subsequent reaction pathways. These effects demonstrate that an organic layer should not be regarded simply as a passive coating. Instead, it functions as a dynamically evolving secondary coordination environment that connects molecular structure with the catalytic state of Cu and the surrounding electrolyte.

A central conclusion of this review is that these mechanisms rarely operate independently. Changes in molecular charge or coordination are commonly accompanied by variations in wettability, ion organization, surface coverage, and intermediate solvation. Likewise, apparent improvements in electronic structure may partly originate from changes in the working-state Cu surface or local mass transport. Organic modifiers should therefore be evaluated as components of a coupled molecule–Cu–electrolyte interface rather than through isolated descriptors alone. Available evidence also suggests that most modifiers do not act as independent catalytic centers capable of completing the full CO_2_RR cycle. Their more general role is to regulate Cu active sites and reaction intermediates through cooperative interfacial interactions.

Despite substantial advances, the field remains at an early stage of rational design. Quantitative relationships among molecular structure, interfacial configuration, working-state evolution, and product selectivity are still incomplete. In addition, the stability of an organic–Cu interface cannot be inferred solely from its initial binding strength because both the molecular layer and the Cu substrate continuously respond to potential, electrolyte composition, adsorbate coverage, and mass-transport conditions. Progress toward practical organic-modified Cu catalysts will therefore require simultaneous control of molecular attachment, dynamic adaptability, catalytic microenvironment, and device-level transport.

### 5.2. Future Perspectives

Future research should move from empirical molecular screening toward quantitative and predictive interface design. A major priority is the establishment of physically meaningful molecular and interfacial descriptors. Parameters such as molecular charge and dipole moment, electron-donating or electron-withdrawing ability, pK_a_, hydrophobicity, steric volume, Cu-binding energy, molecular orientation, surface coverage, and intermolecular interaction strength should be considered together with catalyst descriptors, including Cu facet, defect density, oxidation state, coordination environment, and active-site density. These parameters must also be linked to electrolyte composition, applied potential, current density, and reactor configuration. Curated databases integrating molecular structure, interface characterization, operating conditions, and catalytic performance would support high-throughput computation and machine-learning-assisted screening. Importantly, data-driven models should remain chemically interpretable and be constrained by electrochemical thermodynamics, surface chemistry, and transport physics rather than relying only on statistical correlations.

A second challenge is to determine the actual structure and function of organic–Cu interfaces under reaction conditions. Ex situ characterization often cannot distinguish the initially prepared interface from the catalytically relevant working state. Applied potential may induce molecular reorientation, partial desorption, oligomerization, ion exchange, protonation or deprotonation, and chemical degradation, while Cu simultaneously undergoes redox changes and morphological reconstruction. Time-resolved operando XAS, surface-enhanced infrared and Raman spectroscopy, ambient-pressure photoelectron spectroscopy, electrochemical mass spectrometry, and liquid-cell electron microscopy should therefore be combined to monitor both the organic layer and Cu surface. Isotope labeling is also essential for distinguishing genuine CO_2_-derived products from carbon released through decomposition of organic modifiers. Closely matched control experiments, including modifiers with systematically varied functional groups and molecular structures, will be necessary to separate electronic effects from changes in wettability, local pH, solvation, and mass transport.

Third, the interfacial stability should be treated as a dynamic design problem rather than as the maximization of molecule–Cu binding strength. The weak adsorption leads to rapid molecular loss, whereas an overly rigid layer may block active sites, restrict reactant transport, or fail to accommodate reconstruction of the Cu substrate. Future modifiers should combine sufficiently strong anchoring with controlled flexibility and permeability. Multipoint coordination, cross-linked polymer networks, self-assembled architectures, porous organic frameworks, and poly (ionic liquid)s offer possible routes to such adaptive interfaces. Stability studies should track molecular retention, functional-group integrity, polymer swelling, ion exchange, changes in surface coverage, and reconstruction of Cu rather than reporting catalytic current and Faradaic efficiency alone. Accelerated degradation protocols and quantitative post-electrolysis mass balances would further help identify the dominant failure mechanisms.

Fourth, the design of organic modifiers should evolve from single-function surface modification toward integrated reaction-microdomain engineering. An optimal interface may need to simultaneously regulate the electronic state of Cu, enrich CO_2_, control water and proton availability, organize interfacial ions, stabilize selected intermediates, and facilitate product removal. These requirements may not be satisfied by a single functional group. Hierarchical layers containing different molecular domains could instead provide spatially separated functions. For example, an inner anchoring layer could regulate Cu active sites, an intermediate polar or ionic region could stabilize reaction intermediates and organize the electric field, and an outer porous or hydrophobic region could control CO_2_ transport and suppress excessive water access. Precisely positioned molecular sites may also create tandem microdomains in which CO-generating, CO-retaining, and C–C-coupling functions operate cooperatively. The major challenge will be achieving such multifunctionality without introducing excessive diffusion resistance or blocking catalytically accessible Cu sites.

Fifth, molecular interface design must be integrated with realistic electrolyzer operation. H-cells, flow cells, and membrane-electrode assemblies (MEAs) provide substantially different CO_2_-transport pathways, interfacial environments, and operating regimes. Performance obtained in static H-cells may therefore not be retained in gas-diffusion electrodes, flow cells, or MEAs because organic layers experience markedly different local pH, gas–liquid transport, ion flux, pressure, and mechanical stress. Future studies should therefore evaluate organic-modified Cu catalysts at commercially relevant current densities and over extended continuous operation. In addition to Faradaic efficiency and partial current density, reports should include full-cell voltage, energy efficiency, single-pass carbon efficiency, carbon balance, product concentration, CO_2_ utilization, electrode flooding, salt precipitation, and modifier retention. Standardized reporting of modifier loading, surface coverage, preparation procedures, catalyst area, and post-test characterization would improve comparison among different systems and reduce ambiguity in structure–performance relationships.

Finally, practical implementation will depend on manufacturability, cost, and environmental compatibility. Promising molecular modifiers should be accessible through scalable synthesis, use minimal quantities of expensive or toxic reagents, and remain compatible with catalyst inks, gas-diffusion layers, ionomers, membranes, and continuous electrode-fabrication processes. The environmental impact and possible release of fluorinated molecules, ionic-liquid components, polymer fragments, and metal–organic species should be considered from the beginning of catalyst development. Techno-economic and life-cycle assessments should be coupled with catalytic evaluation to determine whether gains in selectivity and durability justify the added complexity of molecular modification.

Overall, the next stage of this field should establish a direct and predictive connection among molecular structure, working-state interfacial configuration, reaction microenvironment, and device-level performance. Achieving this objective will require coordinated advances in molecular synthesis, operando characterization, theoretical modeling, data science, and electrolyzer engineering. Such an integrated approach could transform organic modification from an empirical surface-treatment strategy into a general platform for constructing selective, stable, and scalable Cu interfaces for CO_2_ conversion to C_2+_ products.

## Figures and Tables

**Figure 1:**
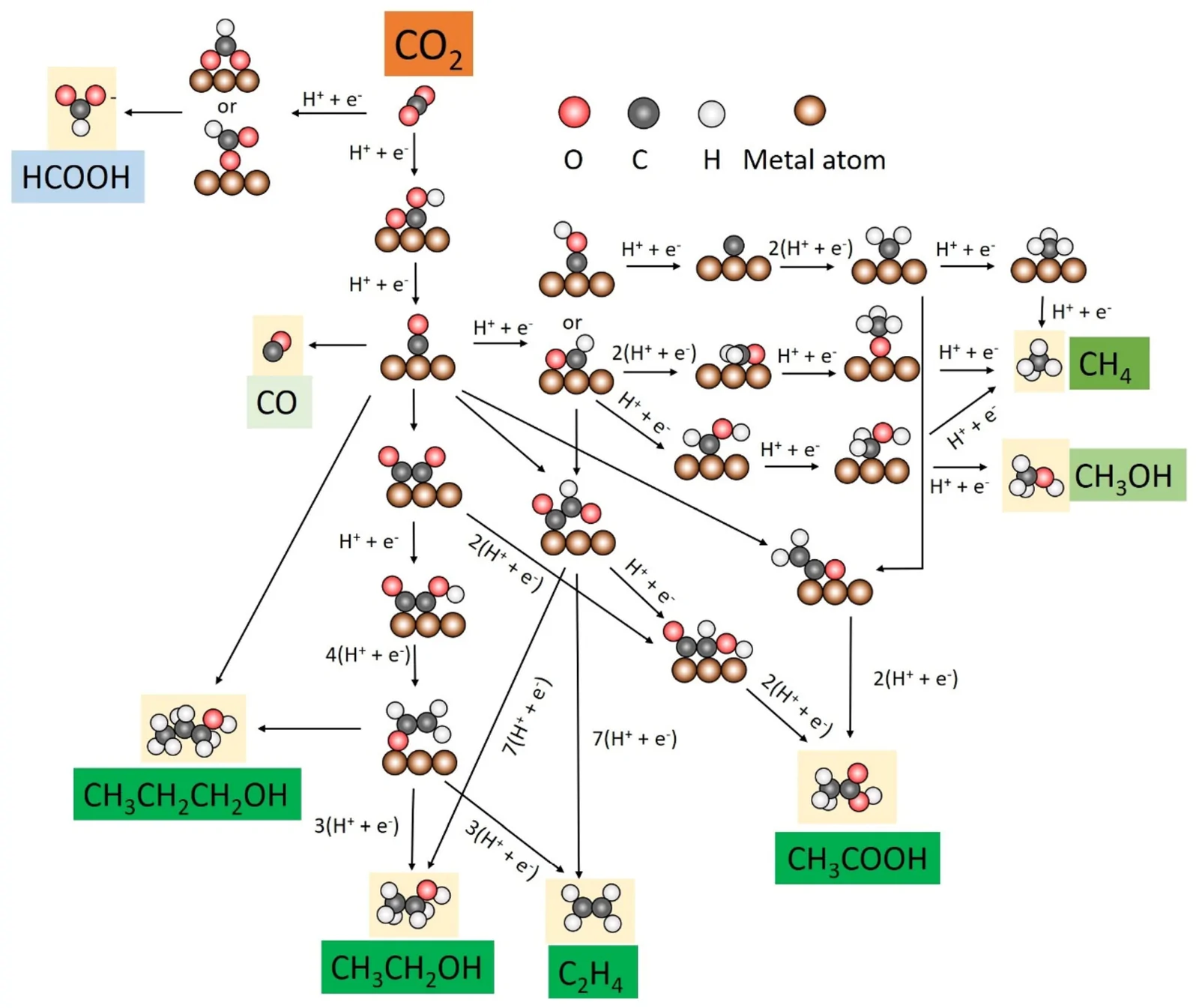
General reaction network for electrochemical CO_2_ reduction toward C_1_ and C_2+_ products on metal surfaces. Reproduced under the terms of CC BY license [39] Copyright 2022, The Authors. MDPI.

**Figure 2:**
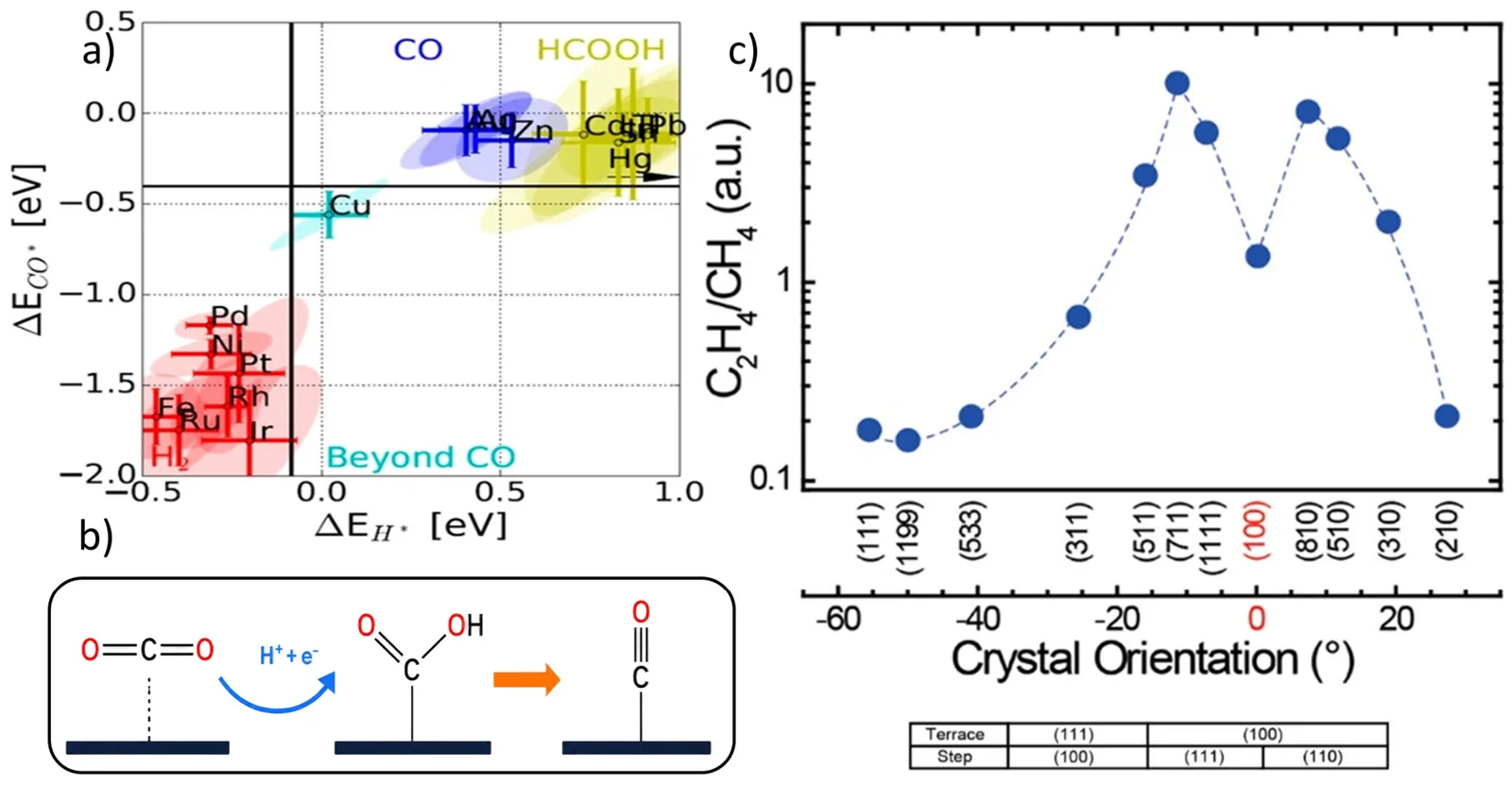
Fundamental reaction characteristics and structure–selectivity relationships for CO_2_ electroreduction on Cu. (**a**) Binding-energy map of CO and H on representative metal surfaces, classifying metals according to their dominant CO_2_RR products and highlighting Cu in the “beyond CO” region. Reproduced with permission from Ref. [40]. Copyright 2017, Wiley-VCH Verlag GmbH & Co. KGaA.; (**b**) Schematic illustration of the initial CO_2_ activation pathway through *COOH formation and subsequent conversion to adsorbed *CO. (**c**) Variation in the C_2_H_4_/CH_4_ selectivity ratio with the crystal orientation relative to Cu (100), illustrating the strong structure dependence of ethylene and methane formation on Cu single-crystal surfaces. Reproduced under terms of the CC BY-NC 4.0 license [2]. Copyright 2019, American Chemical Society.

**Figure 3:**
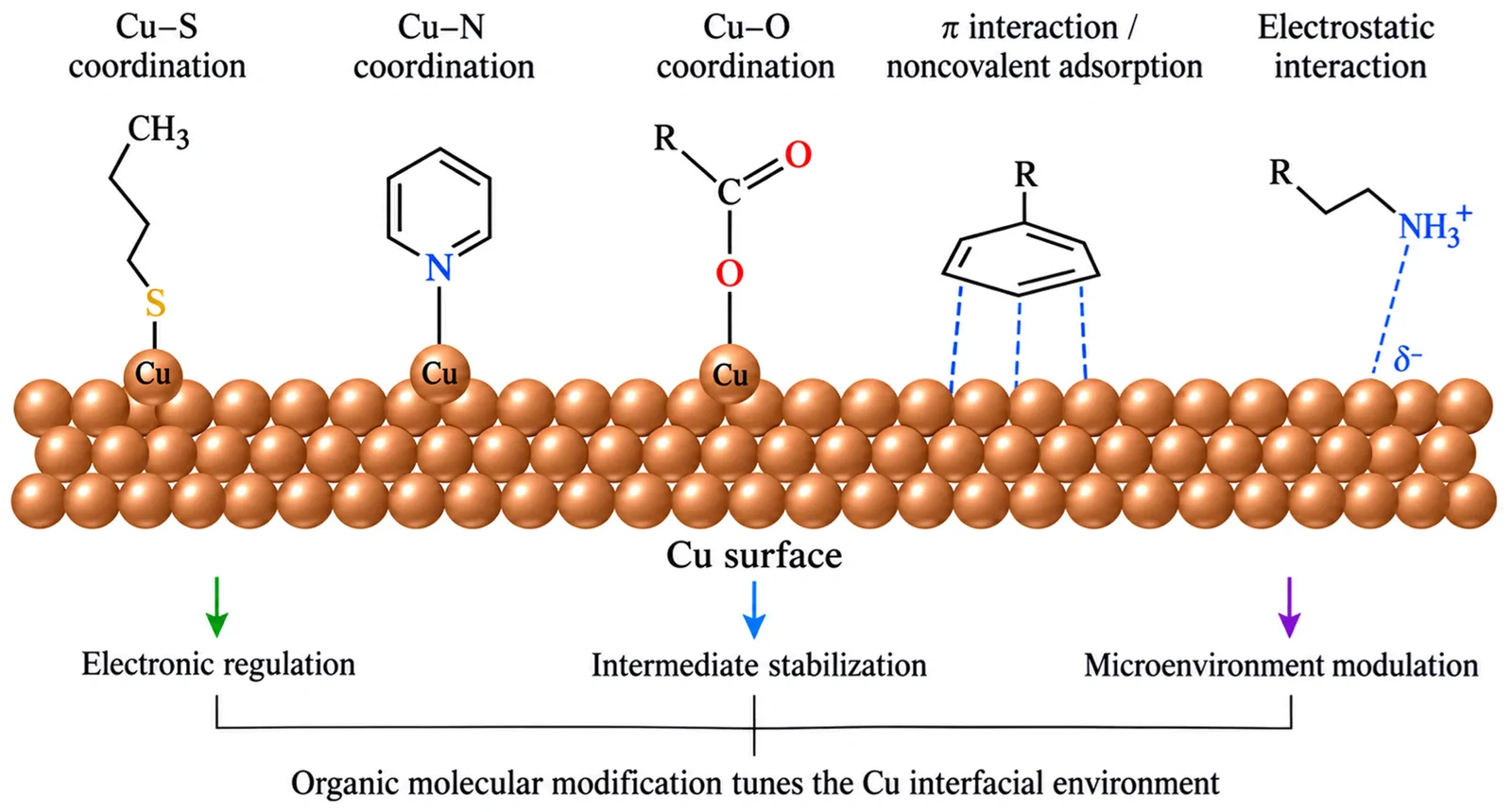
Schematic illustration of representative interaction modes between organic molecules and Cu surfaces.

**Figure 4:**
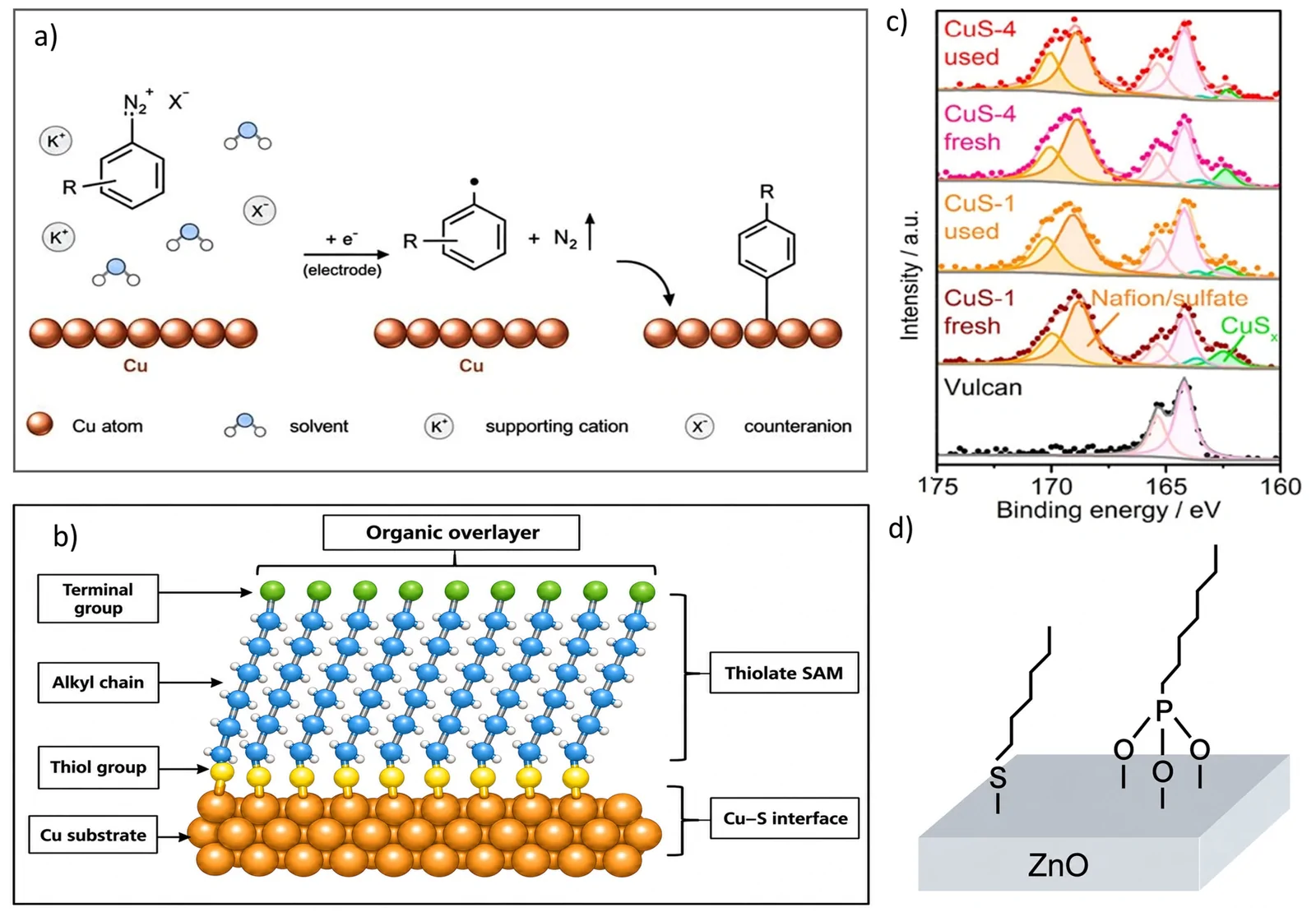
Representative chemical-bonding and coordination motifs relevant to the construction of organic–Cu interfaces. (**a**) Electrochemical grafting pathways of aryldiazonium species and the formation of aryl layers on conductive substrates. (**b**) General architecture and interfacial functions of thiolate self-assembled monolayers on metal surfaces. Orange: Cu atoms; Yellow: S atoms; Blue: C atoms. (**c**) S 2p XPS spectra of sulfur-modified Cu catalysts before and after CO_2_ electrolysis. Reproduced under terms of the CC BY-NC 4.0 license [64]. Copyright 2018, American Chemical Society. (**d**) Comparison of thiol and phosphonic-acid anchoring groups on ZnO as a model for molecular anchoring to oxide surface.

**Figure 5:**
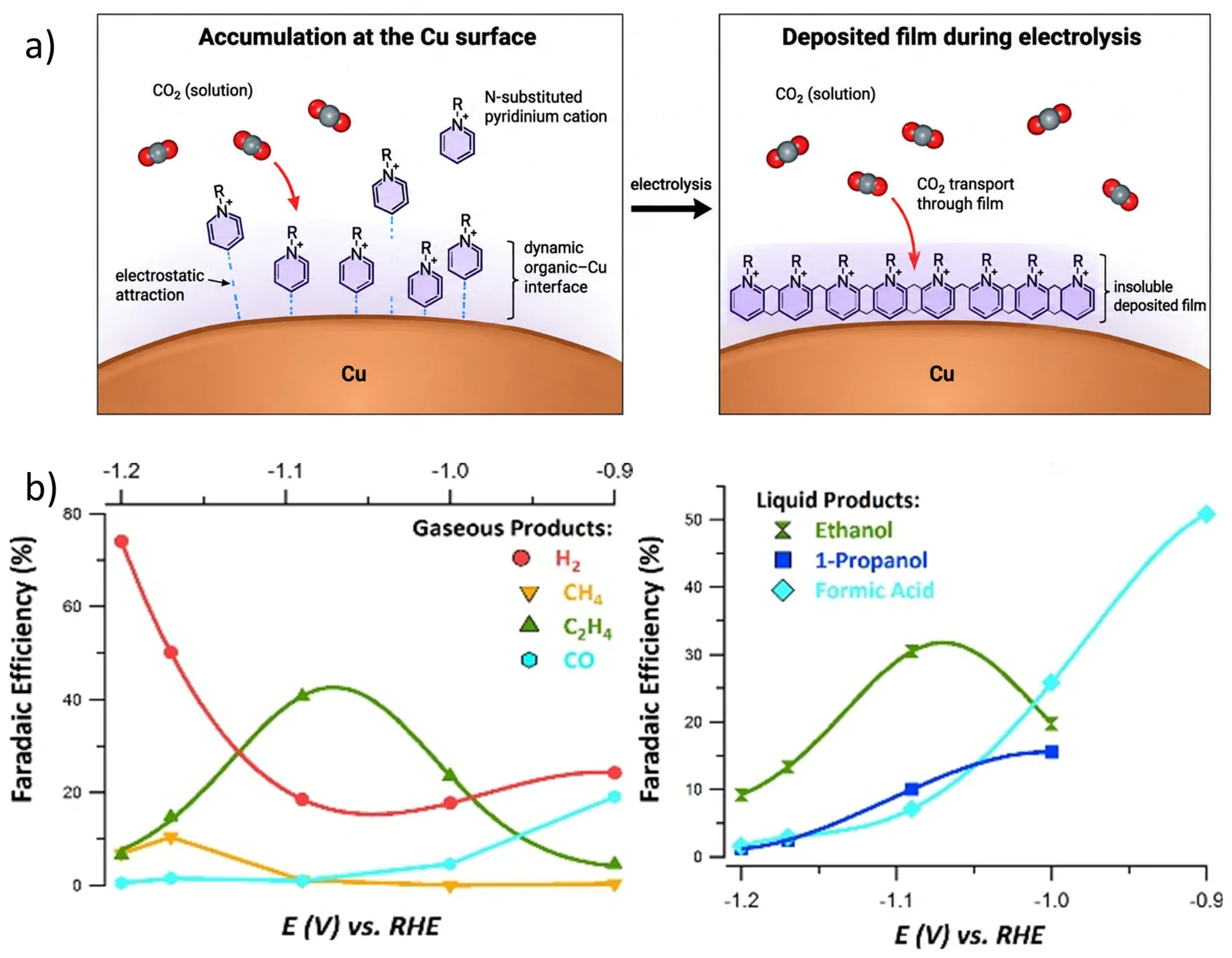
Noncovalently adsorbed and in situ formed organic interfaces relevant to CO_2_RR regulation. (**a**) Schematic comparison of amine- and thiol-capped Ag nanoparticles, illustrating terminal-group-dependent regulation of hydrogen evolution and CO_2_RR-intermediate binding. (**b**) Potential-dependent Faradaic efficiencies of gaseous and liquid products on N-substituted-pyridinium-modified polycrystalline Cu. Reproduced under terms of the CC BY-NC 4.0 license [34]. Copyright 2017, American Chemical Society.

**Figure 7:**
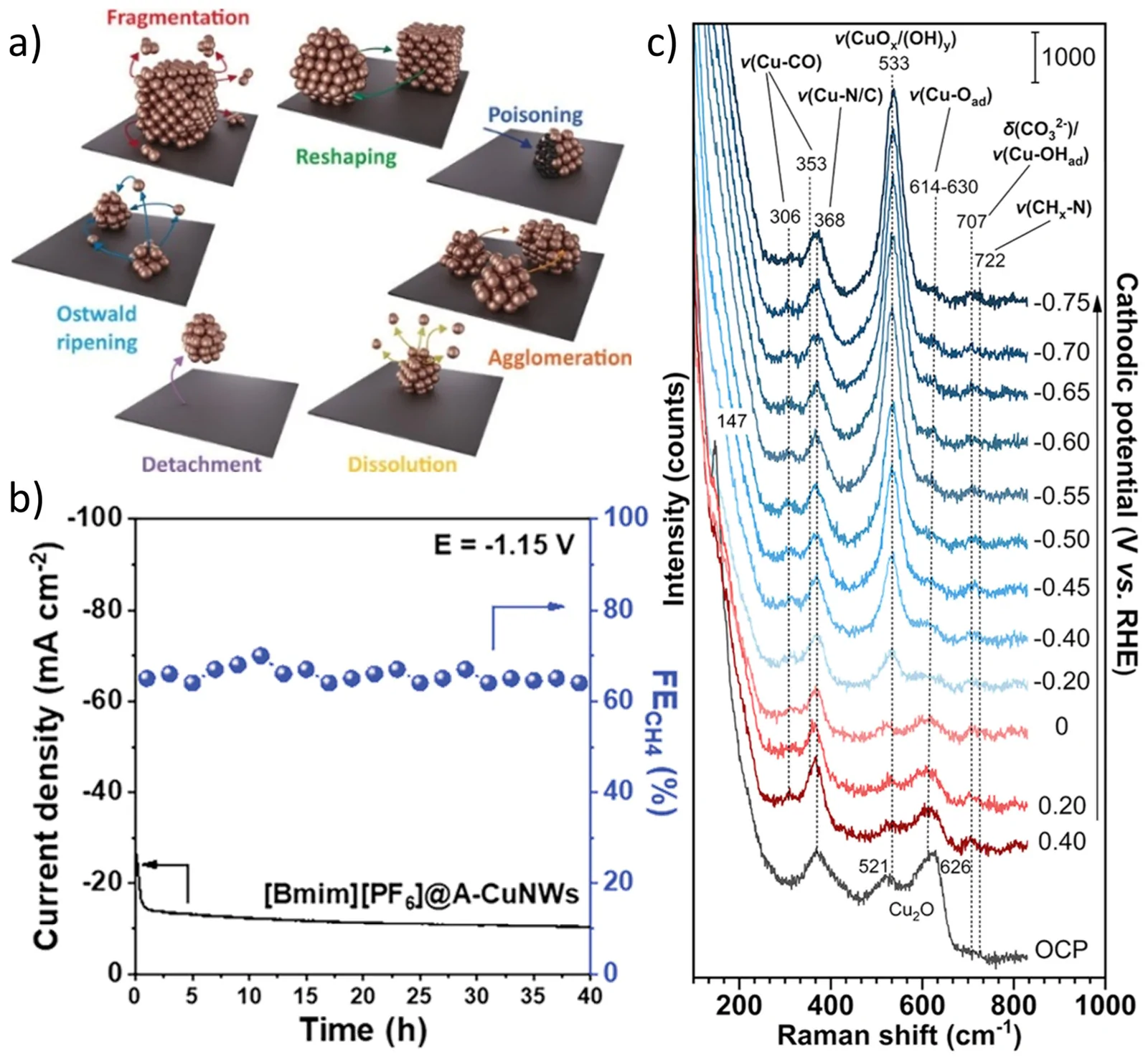
Stability and dynamic evolution of molecule-modified Cu interfaces during CO_2_RR. (**a**) Representative degradation pathways of Cu-based catalysts, including dissolution, detachment, Ostwald ripening, fragmentation, reshaping, poisoning, and agglomeration. Reproduced with permission from Ref. [79]. Copyright 2020, Wiley-VCH GmbH. (**b**) Forty-hour stability of [Bmim][PF_6_]-modified activated Cu nanowires. Reproduced with permission from Ref. [77]. Copyright 2024, Wiley-VCH GmbH. (**c**) Potential-dependent in situ Raman spectra showing the evolution of Cu oxide and Cu–adsorbate features at a poly (ionic liquid)-modified Cu interface. Reproduced with permission from Ref. [78]. Copyright 2022, Wiley-VCH GmbH.

**Figure 8:**
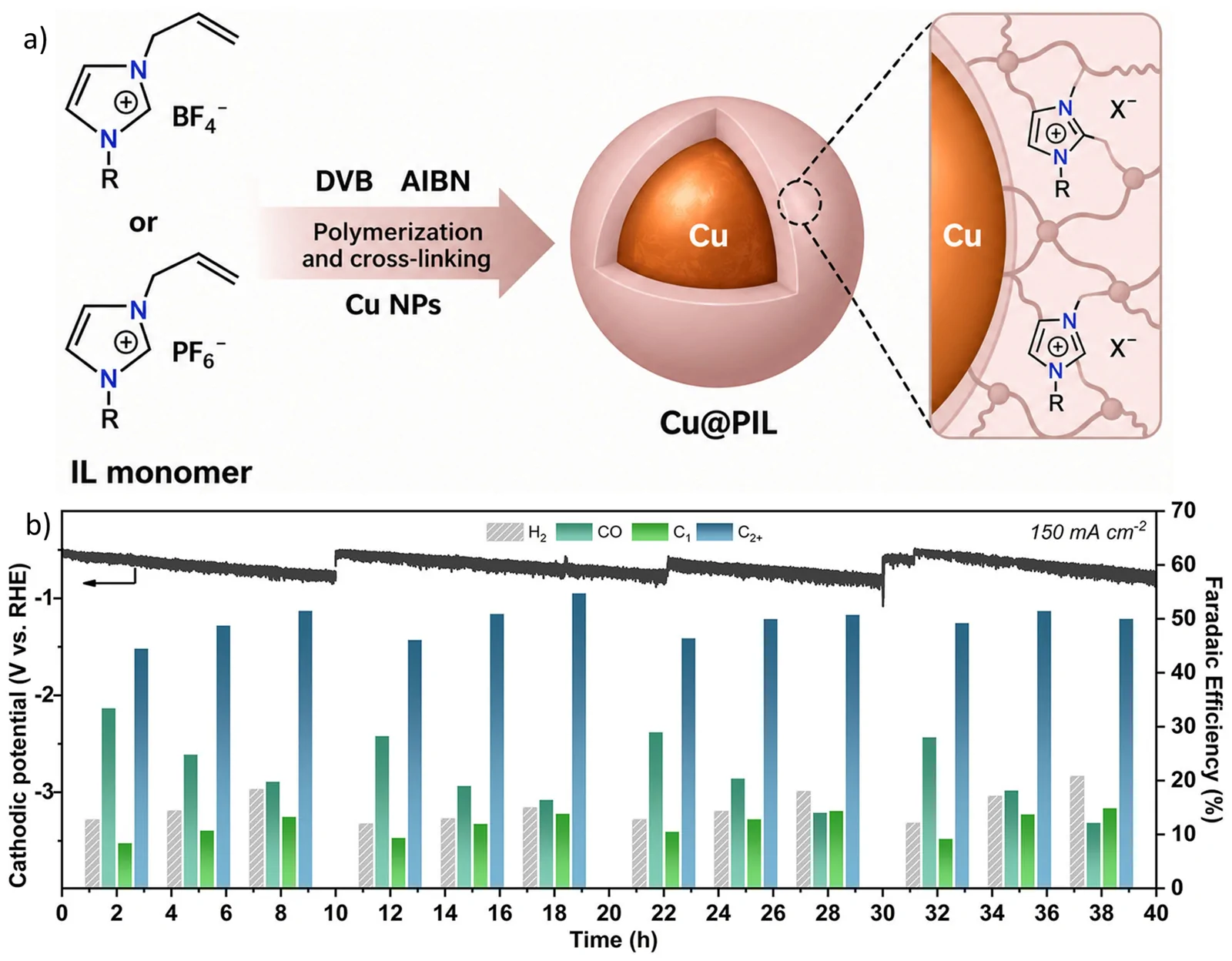
Representative construction strategies and structural features for stabilizing organic–Cu interfaces. (**a**) Construction of a core–shell Cu@poly (ionic liquid) interface through polymerization and cross-linking of ionic-liquid monomers around Cu nanoparticles. Reproduced with permission from Ref. [72]. Copyright 2021, Elsevier B.V. (**b**) Forty-hour electrolysis performance of a poly(ionic liquid)-based Cu^0^–Cu^I^ tandem catalyst at 150 mA cm^−2^. Reproduced with permission from Ref. [78]. Copyright 2022, Wiley-VCH GmbH.

**Figure 9:**
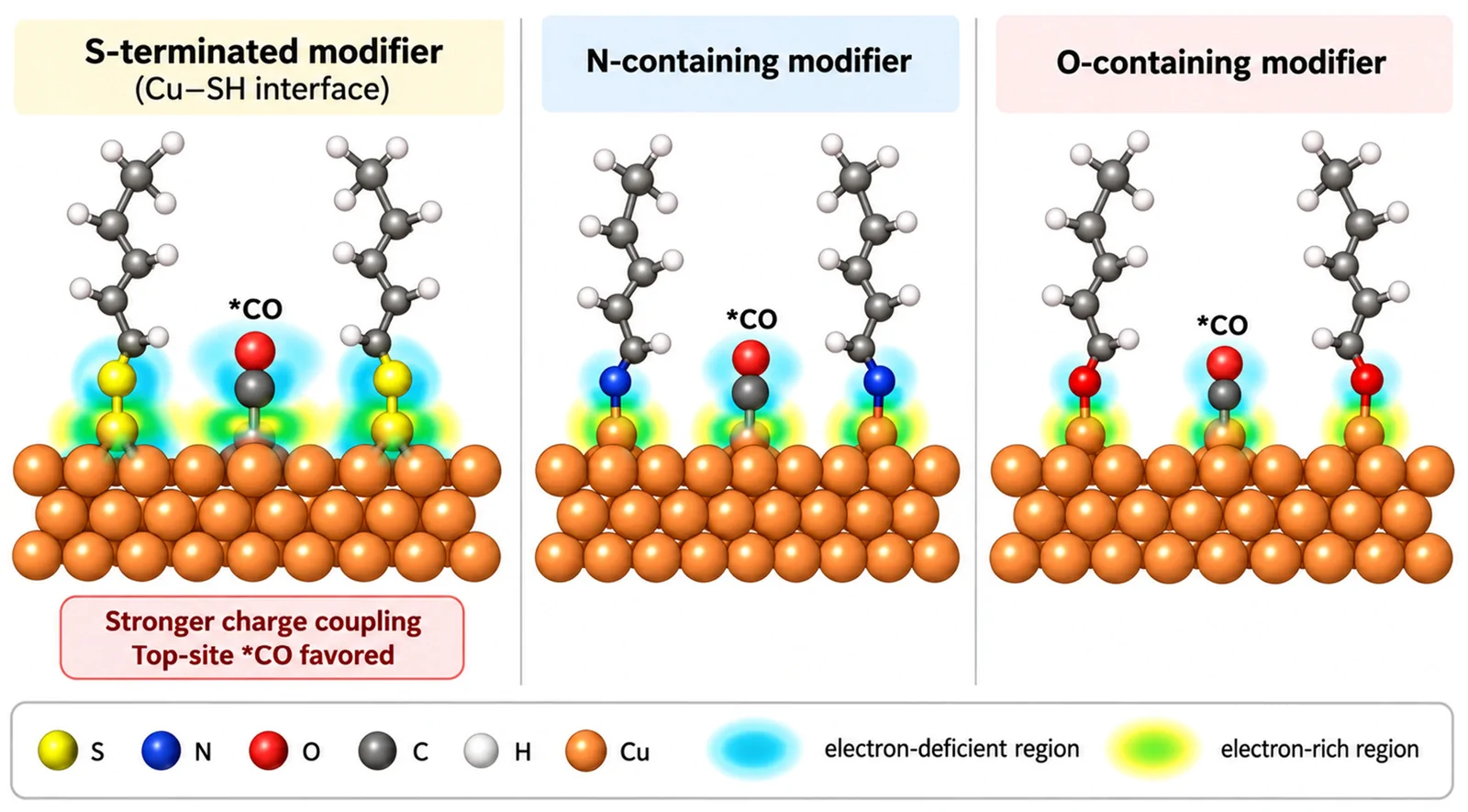
Differential charge-density maps for Cu interfaces modified with S-, N-, and O-terminated long-chain molecules.

**Figure 10:**
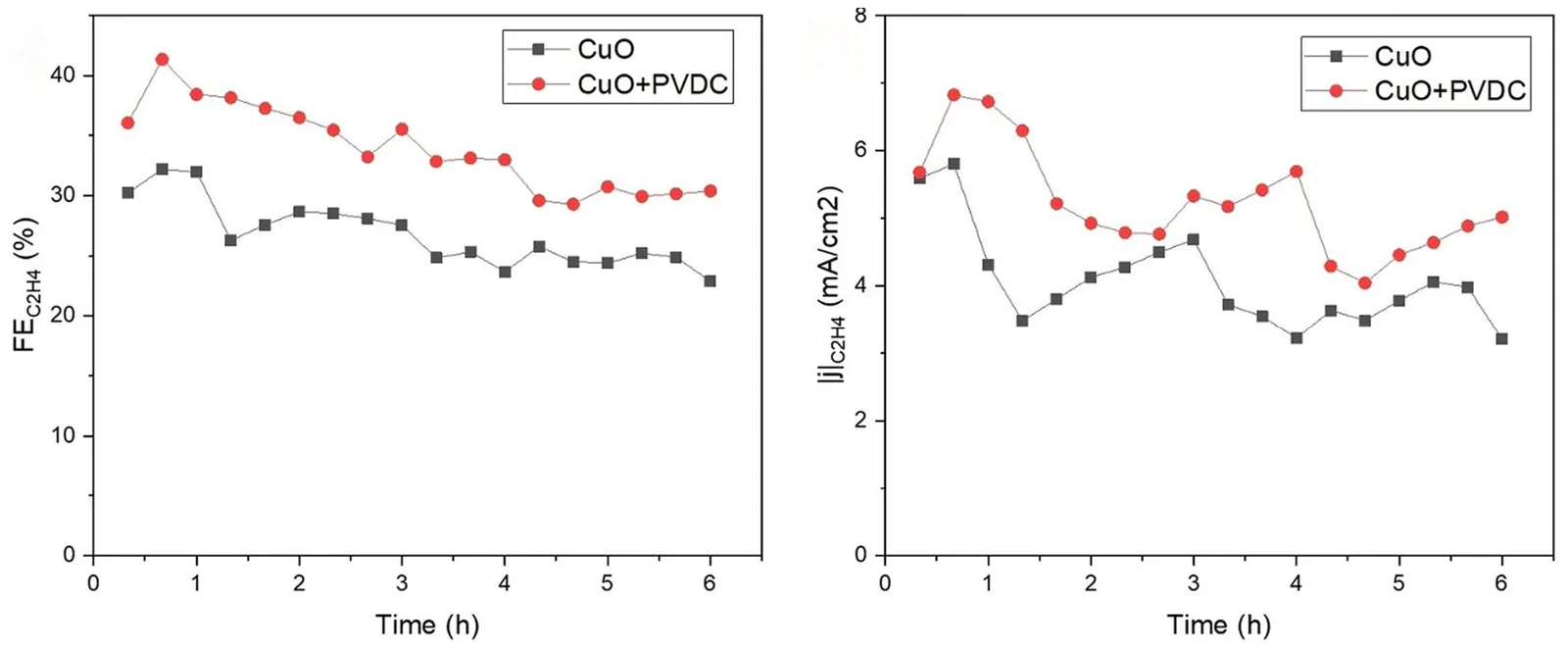
Long-term stability of bare CuO and CuO–PVDC electrodes, showing the evolution of C_2_H_4_ Faradaic efficiency and C_2_H_4_ partial current density during 6 h electrolysis at −0.89 V vs. RHE. Reproduced under terms of CC BY license [89]. Copyright 2024, Elsevier B.V.

**Figure 12:**
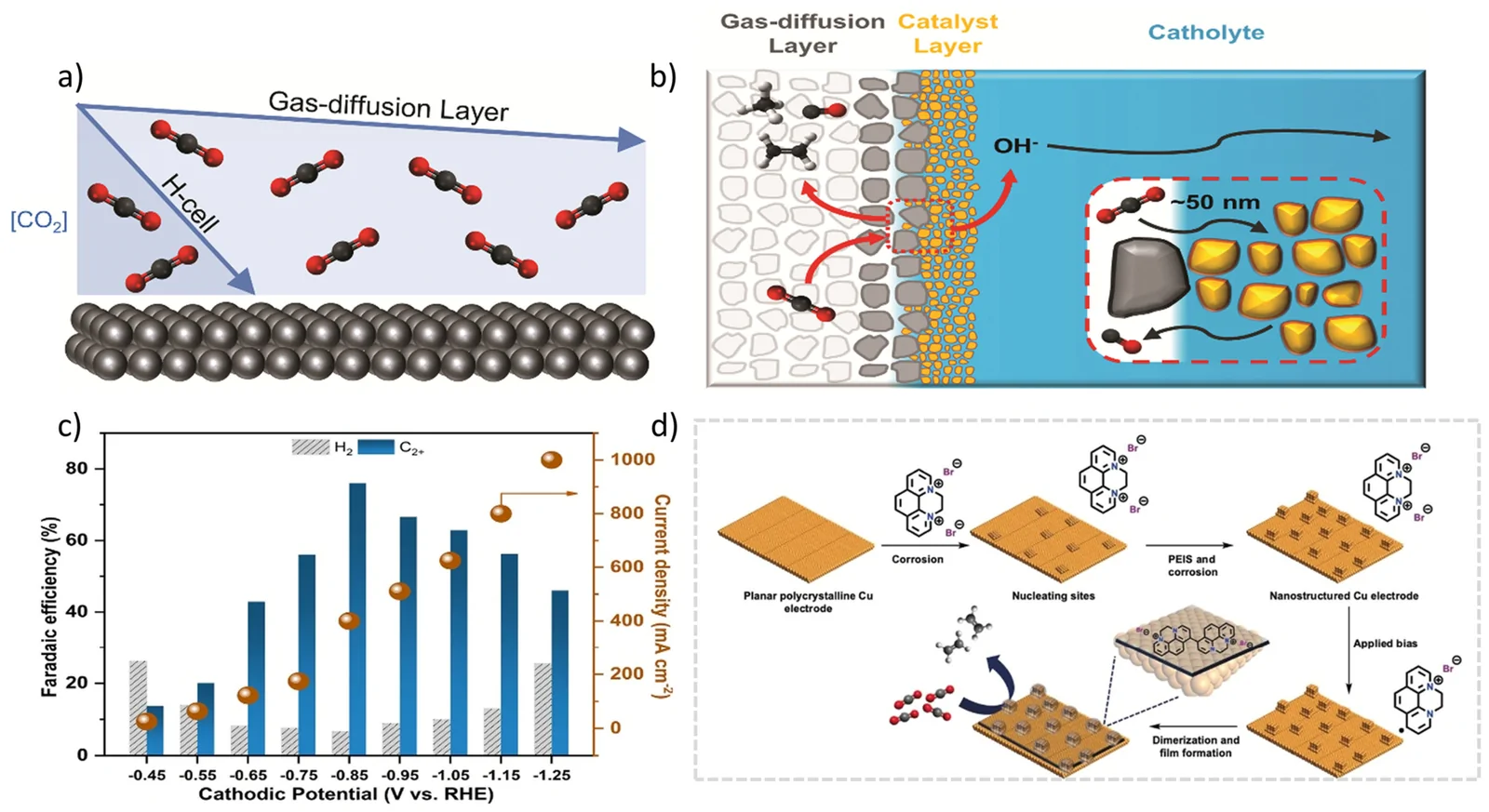
Regulation of CO_2_ availability, interfacial transport, wettability, and electric fields. (**a**) Schematic illustration of the effect of increasing current density on the near-surface CO_2_ concentration, highlighting the different CO_2_-depletion behaviors in H-cell and gas-diffusion-layer configurations. Reproduced under terms of the CC BY 3.0 license [99]. Copyright 2019, The Royal Society of Chemistry. (**b**) Species transport within a catalyst layer deposited on a gas-diffusion electrode, showing the shortened CO_2_ diffusion pathway of approximately 50 nm and the transport of OH^−^ toward the electrolyte or ion-exchange membrane. Reproduced under terms of the CC BY 3.0 license [99]. Copyright 2019, The Royal Society of Chemistry. (**c**) Potential-dependent C_2+_ Faradaic efficiency and current density of a poly (ionic liquid)-based Cu^0^–Cu^I^ tandem catalyst. Reproduced with permission from Ref. [78]. Copyright 2022, Wiley-VCH GmbH. (**d**) In situ nanostructuring and stabilization of polycrystalline Cu induced by an organic-salt additive during CO_2_RR. Reproduced with permission from Ref. [102]. Copyright 2019, Wiley-VCH GmbH.

**Figure 13:**
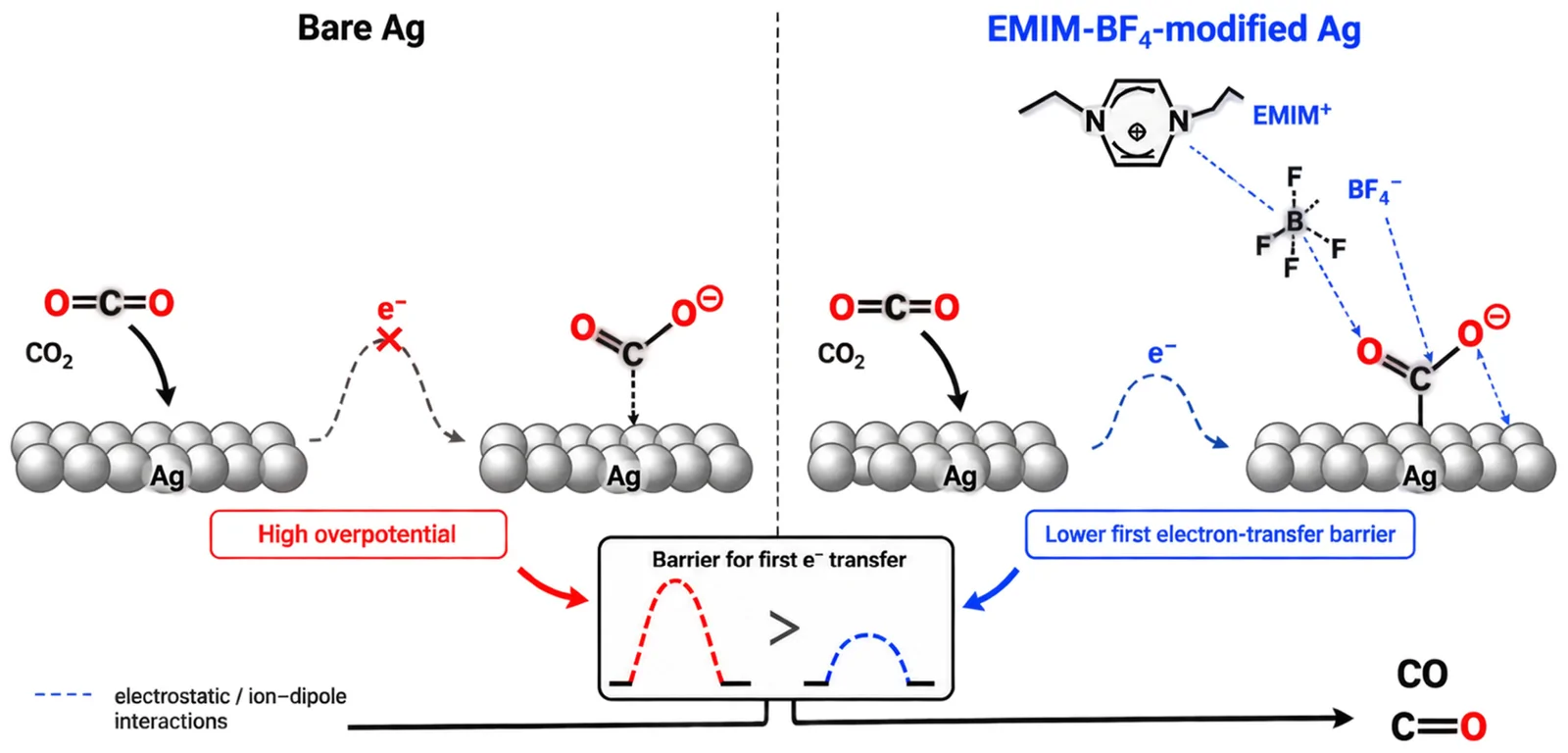
Free-energy profile illustrating the stabilization of an activated CO_2_-derived intermediate by EMIM–BF_4_ on an Ag electrode.

**Figure 14:**
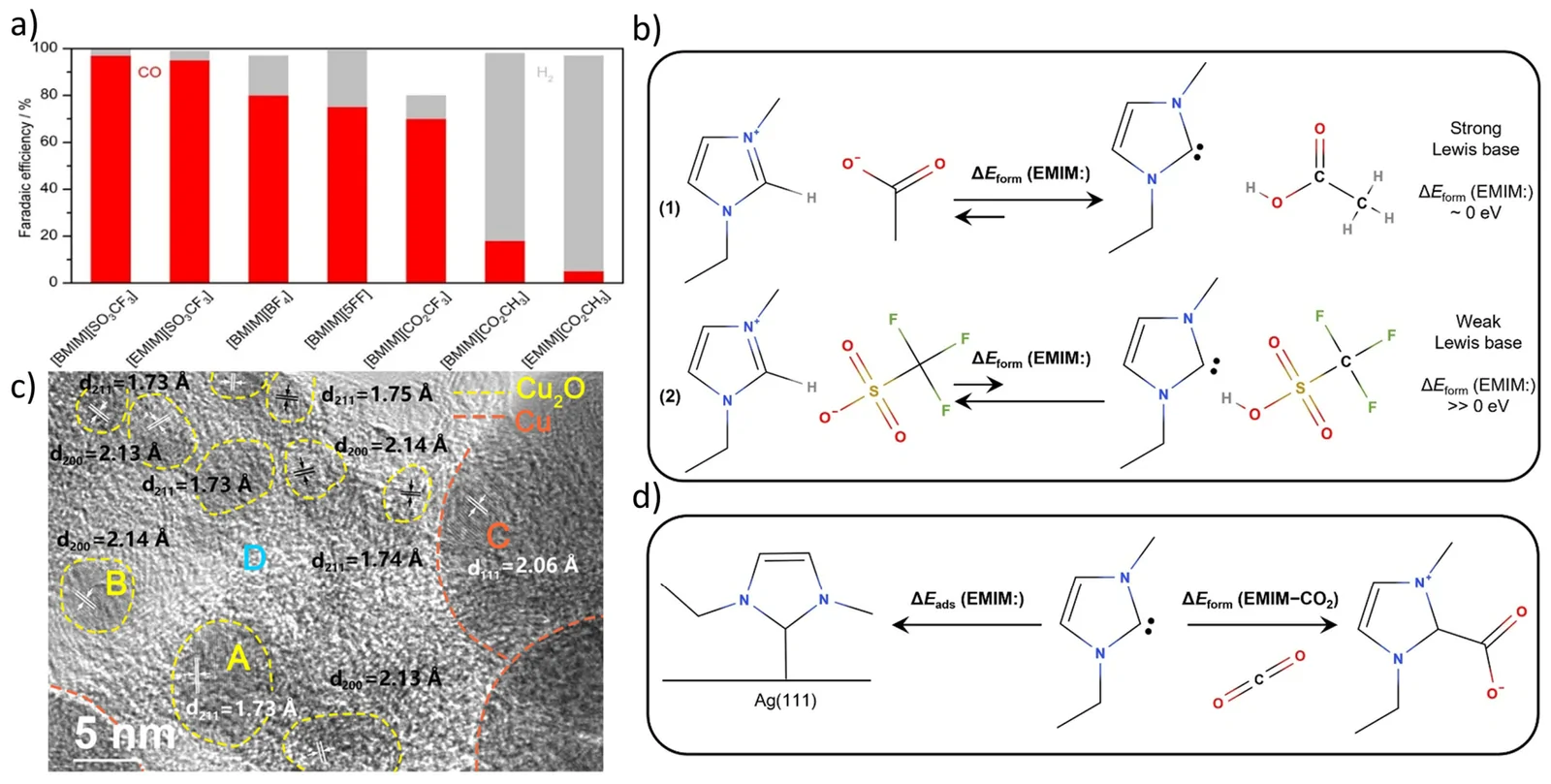
Cooperative roles of ionic-liquid cations, anions, and poly (ionic liquid) frameworks. (**a**) Product Faradaic efficiencies obtained in imidazolium-based electrolytes containing different anions. Reproduced under terms of the CC BY-NC-ND 4.0 license [110]. Copyright 2024, American Chemical Society. (**b**) Influence of strongly (1) and weakly (2) Lewis-basic anions on imidazolium deprotonation and carbene formation. Reproduced under terms of the CC BY-NC-ND 4.0 license [110]. Copyright 2024, American Chemical Society. (**c**) High-resolution transmission electron microscopy image of Cu^0^/Cu_2_O domains confined within a poly (ionic liquid)-based tandem catalyst. Reproduced with permission from Ref. [78]. Copyright 2022, Wiley-VCH GmbH. (**d**) Calculated adsorption and reaction pathways involving EMIM, an N-heterocyclic carbene, and CO_2_ on an Ag (111) model surface. Reproduced under terms of the CC BY-NC-ND 4.0 license [110]. Copyright 2024, American Chemical Society.

## Data Availability

The original contributions presented in this study are included in the article. Further inquiries can be directed to the corresponding author.
